# Cryptic splicing of stathmin-2 and UNC13A mRNAs is a pathological hallmark of TDP-43-associated Alzheimer’s disease

**DOI:** 10.1007/s00401-023-02655-0

**Published:** 2024-01-04

**Authors:** Ana Rita Agra Almeida Quadros, Zhaozhi Li, Xue Wang, I. Sandra Ndayambaje, Sandeep Aryal, Nandini Ramesh, Matthew Nolan, Rojashree Jayakumar, Yi Han, Hannah Stillman, Corey Aguilar, Hayden J. Wheeler, Theresa Connors, Jone Lopez-Erauskin, Michael W. Baughn, Ze’ev Melamed, Melinda S. Beccari, Laura Olmedo Martínez, Michael Canori, Chao-Zong Lee, Laura Moran, Isabelle Draper, Alan S. Kopin, Derek H. Oakley, Dennis W. Dickson, Don W. Cleveland, Bradley T. Hyman, Sudeshna Das, Nilüfer Ertekin-Taner, Clotilde Lagier-Tourenne

**Affiliations:** 1 Department of Neurology, The Sean M. Healey and AMG Center for ALS, Massachusetts General Hospital, Harvard Medical School, Boston, MA USA; 2 Department of Neurology, MassGeneral Institute for Neurodegenerative Diseases (MIND), Massachusetts General Hospital, Harvard Medical School, Boston, MA USA; 3 Broad Institute of Harvard University and MIT, Cambridge, MA USA; 4 Department of Quantitative Health Sciences, Mayo Clinic, Jacksonville, FL USA; 5 Department of Pathology, Massachusetts General Hospital, Harvard Medical School, Boston, MA USA; 6 Department of Cellular and Molecular Medicine, University of California at San Diego, La Jolla, CA USA; 7 AUTTX LLC, Wellesley, MA USA; 8 Department of Neuroscience, Mayo Clinic, Jacksonville, FL USA; 9 Department of Neurology, Mayo Clinic, Jacksonville, FL USA

**Keywords:** Stathmin-2, SCG-10, UNC13A, TDP-43, TARDBP, Alzheimer’s disease, Cryptic exons

## Abstract

Nuclear clearance and cytoplasmic accumulations of the RNA-binding protein TDP-43 are pathological hallmarks in almost all patients with amyotrophic lateral sclerosis (ALS) and up to 50% of patients with frontotemporal dementia (FTD) and Alzheimer’s disease. In Alzheimer’s disease, TDP-43 pathology is predominantly observed in the limbic system and correlates with cognitive decline and reduced hippocampal volume. Disruption of nuclear TDP-43 function leads to abnormal RNA splicing and incorporation of erroneous cryptic exons in numerous transcripts including Stathmin-2 (*STMN2*, also known as *SCG10*) and *UNC13A*, recently reported in tissues from patients with ALS and FTD. Here, we identify both *STMN2* and *UNC13A* cryptic exons in Alzheimer’s disease patients, that correlate with TDP-43 pathology burden, but not with amyloid-β or tau deposits. We also demonstrate that processing of the *STMN2* pre-mRNA is more sensitive to TDP-43 loss of function than *UNC13A*. In addition, full-length RNAs encoding *STMN2* and *UNC13A* are suppressed in large RNA-seq datasets generated from Alzheimer’s disease post-mortem brain tissue. Collectively, these results open exciting new avenues to use *STMN2* and *UNC13A* as potential therapeutic targets in a broad range of neurodegenerative conditions with TDP-43 proteinopathy including Alzheimer’s disease.

**Supplementary Information:**

The online version contains supplementary material available at 10.1007/s00401-023-02655-0.

## Introduction

Alzheimer’s disease is a progressive neurodegenerative disorder, affecting up to 30% of the population over 65 [59]. Genetic and pathological studies have provided overwhelming evidence that Alzheimer’s disease has a heterogeneous etiology, thus a better characterization of patient subgroups is essential. Extracellular deposits of amyloid-β plaques, and intraneuronal accumulations of neurofibrillary tau tangles are canonical hallmarks of Alzheimer’s disease pathology [14]. Cytoplasmic inclusions of phosphorylated TAR DNA-binding protein-43 (TDP-43) are a pathological hallmark of several neurodegenerative disorders including amyotrophic lateral sclerosis (ALS), frontotemporal dementia (FTD) and limbic-predominant age-related TDP-43 encephalopathy (LATE) [6, 20, 48, 53, 66–68, 81]. Notably, TDP-43 proteinopathy has also been reported in approximately one-third of Alzheimer’s disease cases, with estimates varying from 19 to 57% [5, 7, 13, 21, 29, 34, 35, 38, 54, 85, 92, 100], also referred to as Alzheimer’s disease with LATE neuropathologic changes (LATE-NC) [65–67]. TDP-43 pathology burden in Alzheimer’s disease follows a stereotypic spread, starting in the amygdala and entorhinal cortex, with progression through stages that correlates with a decrease in cognition and hippocampal volume [32–34]. Moreover, TDP-43 pathology in Alzheimer’s disease is associated with loss of cognitive resilience, higher odds of clinical dementia diagnosis [17, 18, 32, 36, 37, 99], and faster brain atrophy rates [33]. While disruption of TDP-43 defines a distinct subgroup of Alzheimer’s disease patients, the impact of TDP-43 pathology and its associated loss of function remains largely unexplored in this condition.

TDP-43 is an RNA-binding protein encoded by the *TARDBP* gene that regulates fundamental aspects of RNA metabolism including splicing [20, 48, 49, 53, 71, 74, 90]. In normal conditions, TDP-43 is mainly nuclear and represses the inclusion of mostly non-conserved cryptic exons [52]. In disease, TDP-43 is cleared from the nucleus and accumulates in phosphorylated and poly-ubiquitinated cytoplasmic inclusions [6, 68]. Loss of TDP-43 nuclear function leads to the aberrant incorporation of cryptic exons into messenger RNAs, which are detectable in tissues from patients with ALS [16, 42, 52, 57, 62], FTD [16, 57, 76], and Alzheimer’s disease [22, 87]. We and others have identified that the human RNA most affected by TDP-43 disruption encodes a neuronal protein called stathmin-2 (STMN2, also known as SCG10) [42, 62]. Indeed, TDP-43 is required to prevent the inclusion of a cryptic exon within the first intron of *STMN2* pre-mRNA. Abnormal inclusion of this cryptic exon and usage of a premature polyadenylation signal produces a non-functional truncated mRNA, leading to a striking loss of stathmin-2 protein [10, 42, 62]. Misprocessing of *STMN2* is detected in post-mortem tissues of patients with sporadic and familial forms of ALS and FTD [42, 62, 76]. Remarkably, disruption of *STMN2* mRNA is critical for neuronal dysfunction caused by TDP-43 nuclear loss-of-function. Restoring STMN2 protein levels either by lentivirus-mediated expression of STMN2 [62] or by antisense oligonucleotides (ASO)-mediated steric blockage of *STMN2* misprocessing [10] rescues axonal regeneration and transport defects in iPSC derived human motor neurons depleted for TDP-43 [10, 42, 62]. In addition, germline deletion of *Stmn2* in mice [26, 45, 50] or chronic reduction of *Stmn2* in the nervous system of otherwise normal adult mice [56] leads to motor deficits with denervation of the neuromuscular junctions that recapitulate clinical manifestations observed in patients with ALS. Hence, restoration of stathmin-2, including with ASOs that sterically block the abnormal splicing [10], emerges as an attractive therapeutic strategy in TDP-43 proteinopathies. Recently the transcript encoding UNC13A, a synaptic protein essential for neurotransmission [8, 95], was also shown to be mis-spliced in ALS and FTD [16, 57]. Loss of TDP-43 leads to the abnormal inclusion of a cryptic exon between exon 20 and 21 of the *UNC13A* transcript that results in a premature stop codon and nonsense-mediated decay of *UNC13A* RNA. Interestingly, non-coding polymorphisms within the *UNC13A* gene previously associated with an increased risk of ALS [93] were shown to reduce the affinity of TDP-43 for *UNC13A* RNA and perturb splicing repression of this intronic region [16, 57].

Here, we titrated different levels of TDP-43 downregulation within human neuroblastoma cells to determine the sensitivity of *STMN2* and *UNC13A* pre-mRNA processing to TDP-43 loss. We also show, that both *STMN2* and *UNC13A* RNAs are mis-spliced in the amygdala and entorhinal cortex of a substantial fraction of patients with Alzheimer’s disease. We demonstrate, for the first time, that accumulation of *STMN2* and *UNC13A* cryptic exons correlates with TDP-43 pathology in Alzheimer’s disease, independently of amyloid-β or tau pathological burden. Similarly, our analysis of large RNA-seq datasets from post-mortem tissues also unravels the disruption of *STMN2* and *UNC13A* in Alzheimer’s disease patients. These results highlight the potential of TDP-43-dependent splicing alterations as molecular markers to subgroup Alzheimer’s disease patients. Importantly, ongoing efforts to develop therapeutic strategies preventing TDP-43-associated splicing defects [10] may benefit patients with a range of neurological conditions including ALS, FTD and Alzheimer’s disease.

## Materials and methods

### RNA extraction and complementary DNA synthesis

Total RNA was extracted from 20 to 40 mg of frozen post-mortem brain tissue, or neuroblastoma SH-SY5Y cells, and lysed using Trizol reagent (ThermoFisher, Cat# 10,296,010). Post-mortem frozen brain samples were obtained from the Massachusetts Alzheimer’s Disease Research Center Neuropathology Core operated under the Massachusetts General Hospital IRB guidelines. A mechanical tissue homogenizer was used for brain tissue. RNA was then purified using Phase Lock Gel Heavy tubes (QuantaBio, Cat# 10847-802) and Isolate RNA Mini Kit (Bioline, Cat#BIO-52073). RNA concentration and quality were measured using nanodrop and 750–1000 ng of total RNA used for cDNA synthesis, using High-Capacity cDNA Reverse Transcription Kit (ThermoFisher, Cat# 4374966) according to the manufacturer’s instructions.

### RT-PCR and quantitative PCR

Reverse transcription polymerase chain reactions (RT-PCRs) were performed using Q5 High-Fidelity DNA polymerase (New England BioLabs, Cat# M0492S) according to the manufacturer’s instructions. Quantitative real-time PCR (qRT-PCR) was performed using iTaq Universal SYBR Green (Bio-rad, Cat#1725121), or Taq™ Universal Probes Supermix (Bio-rad, Cat#1725131). In order to increase specificity of the signal, an optimized combination of primers and probes were used for both STMN2 and UNC13A cryptic exons. Probes and primers used for these reactions are listed in Supplementary Table 1. Reactions were run in triplicates in CFX96 real-time PCR machine (Biorad). For analysis, target RNA levels were normalized to the RNA levels of GAPDH (reference transcript) and to Alzheimer’s disease patients. As *STMN2* and *UNC13A* cryptic exons are normally not expressed, certain samples did not display any amplification of these aberrant transcripts. A sample was considered positive for cryptic exons when there was product amplification in all 3 triplicates evaluated.

### SH-SY5Y cell culture and siRNA treatment

SH-SY5Y human neuroblastoma cells (ATCC Cat# CRL-2266) were cultured in DMEM F12 (Gibco, Cat#1132-033) containing 1% of Penicillin–Streptomycin (Gibco, Cat#15140-122) and 10% of Fetal Bovine Serum (Sigma, Cat# F0926) and kept at 37 °C and 5% CO_2_. TDP-43 knockdown was achieved by transfecting cells with pooled siRNAs against *TARDBP* (ON-TARGETplus siRNA, Dharmacon, Cat# L-012394) or control siRNA (ON-TARGETplus Non-targeting Control Pool, Dharmacon, Cat# D001810–10) using Lipofectamine RNAiMAX Transfection Reagent (Cat# 13778075) in Opti-MEM™ (Gibco, Cat#31985070). For the dose-dependency analyses we used decreasing amounts of siRNAs ranging from 25 to 0.125 pmol per well. Cells were treated with siRNA either for 3 or 5 days, as previous studies reported decrease in *UNC13A* transcripts after longer knockdown of TDP-43 [16, 57].

### Generation of genetically modified SH-SY5Y cells

Cells containing the N352S mutation in both TDP-43 alleles were previously reported [62]. In short, a plasmid containing a single guide RNA (GCGGGTAATAACCAAAACCA) was electroporated in SH-SY5Y cells using a Amaxa Nucleofector (assay A-023), along with a 180-nucleotide long single-stranded donor oligonucleotides (IDT), containing the desired ALS-causing mutation and four synonymous single-nucleotide replacements to avoid DNA cleavage recurrence by Cas9. Cells were sorted and single-cell seeded into a 96-well plate to obtain individual clones. Replacement of AAT to AGT (c.1055A > G) was confirmed by Sanger sequencing.

To obtain cells overexpressing heterogeneous nuclear ribonucleoprotein L (hnRNP L), SH-SY5Y cells were treated with polybrene (Santa Cruz, Cat# sc-134220) and transduced with lentiviral particles encoding the HNRNP L transcript (Origene Cat# CW307794V) or a GFP control (Origene Cat# CW307797V). Transduced cells were then selected using 1 μg/mL puromycin (ThermoFisher, Cat#A1113803). Overexpression of hnRNP L was verified using qRT-PCR (Forward 5′-GTGGAGATGGCTGATGGCTAC-3′, Reverse 5′ GCTCATCGCAGATCTCAAAGAAG-3′) and Western blot (Antibody Sigma Cat#R4903) with GFP overexpressing cells used as control. SH-SY5Y cells were transfected using lipofectamine with 2.5 pMol of TDP-43 or control siRNAs and harvested 3 days later for qRT-PCR analysis.

### Cell toxicity assay

Cell toxicity was assessed using the CellTox Green Toxicity assay (Promega, Cat#G8742) according to manufacturer’s instructions. Cells were seeded in a 96-well plate, and toxicity measured after 5 days of siRNA treatment. Cells were treated with CellTox Green Reagent for 15 min at room temperature and shielded from light. As positive control, the cells were lysed for 30 min using the lysis buffer provided by the Promega. Fluorescence was measured on a Synergy2 plate reader (BioTek Instruments) using 485 nm excitation and 528 nm emission.

### Immunostaining of post-mortem tissues and image analysis

Formalin fixed paraffin-embedded brain sections from control or Alzheimer’s disease patients were obtained from the Massachusetts Alzheimer’s Disease Research Center Neuropathology Core operated under the Massachusetts General Hospital IRB guidelines. Brains donated to the Massachusetts Alzheimer’s Disease Research Center, are cut in half, one hemisphere is fresh frozen while the contralateral hemisphere is fixed in 10% neutral buffered formalin. The fixed hemisphere was sectioned and an average of 19–22 distinct regions of interest were mounted in blocks for histological examination. Alzheimer’s disease patients were classified using clinical and neuropathological criteria [30], and aged and sex matched control subjects were chosen that had no significant clinical or neuropathological abnormalities. Demographic characteristics of Alzheimer’s disease patients and control individuals selected are described in Supplementary Table 2. The cohort of patients in this study did not include any patient with pure LATE-NC (i.e. without Alzheimer’s disease neuropathological changes).

For immunohistochemistry and immunofluorescence of TDP-43, sections were first dehydrated in an oven for 1 h at 70 °C, followed by sequential steps of deparaffinization and rehydration using Citrosolv (Decon Laboratories, Cat# 04-355-121), 100% Ethanol (Fisher Scientific, Cat# BP28184), 90% Ethanol, 70% Ethanol and demineralized water. Antigen retrieval was performed using Sodium Citrate Buffer (Fisher Scientific, Cat#S279-500) pH 6.0 for 20 min at 120 °C. Endogenous peroxidases were quenched with a treatment of 3% H_2_O_2_ (Millipore Sigma, Cat# HX0635) for 30 min. Sections were blocked for one hour in a solution of 1:10 donkey serum (Jackson Immuno Research Labs, Cat#NC9624464) in TBS-0.1% Triton (Tris Buffered Saline, Boston BioProducts, Cat# BM-301; Triton Sigma, Cat#T8787). Sections were incubated overnight at 4 °C with phosphorylated TDP-43 primary antibody (CosmoBio Mouse Polyclonal, Cat#CAC-TIP-PTD-M01, 1:10,000), or TDP-43 primary antibody (Proteintech, Rabbit Polyclonal, Cat# 10782-2-AP, 1:500) diluted in blocking solution. The next day sections were washed 3× using TBS-0.1% Triton, and then incubated with corresponding secondary antibodies for 2 h at room temperature. For immunohistochemistry the EnVision + Single Reagent HRP Mouse (Agilent, Cat# K400111-2) system was used according to manufacturer’s instructions, and cell nuclei were stained for 30 s using Hematoxylin (Fisher Scientific, Cat#3530-32). For immunofluorescence, slides were incubated with Alexa Fluor anti-rabbit 488 and Alexa Fluor anti-mouse 568 secondary antibodies, and cell nuclei were stained using DAPI. Finally, slides were dehydrated by a sequential treatment with demineralized water, 70% Ethanol, 90% Ethanol, 100% Ethanol and Citrosolv, and then coverslipped with Epredia mounting media (Epredia, Cat#4112). At the end, immunofluorescent slides were treated with 0.1% Sudan Black, to quench the human tissue auto-fluorescence. Total tau (1:6000 dilution, Agilent/Dako, #A002401-2, 1), and amyloid-β 6F/3D (1:600 dilution, Agilent/Dako, #M087201-2) were stained using a Leica BOND-RX automated stainer.

For immunohistochemistry, whole-slide images were taken using brightfield 40× objective (NanoZoomer, Hamamatsu), and a confocal Nikon C2 Confocal was used to image slides stained by immunofluorescence at a 60× magnification. To quantify the percentage of area occupied by phosphorylated TDP-43, Tau or amyloid-β, five images of the same area were selected within the amygdala for each slide and extracted for quantification using FIJI [82]. The Color Threshold and Analyze particles plugins from FIJI were used to quantify the area stained for each protein. Investigators were blinded to the disease status during the experimental procedure and analysis.

TDP-43 staging was determined according to the simplified staging method proposed by Nelson et al. [67]. In short, patients were classified according to the presence of TDP-43 proteinopathy in the amygdala (stage I), hippocampus (stage II) and frontal cortex (stage III). The information obtained for each individual is included in Supplementary Table 3.

### Bioinformatics analysis of RNA-seq data

The Mayo Clinic RNA-seq cohort has been made available to the research community and described in detail previously [2, 3, 98]. In brief, Alzheimer’s disease and control subjects were diagnosed neuropathologically at autopsy [61]. Alzheimer’s disease subjects from the Mayo Clinic Brain Bank had definite neuropathologic diagnosis according to the National Institute of Neurological and Communicative Disorders and Stroke and the Alzheimer’s Disease and Related Disorders Association (NINCDS–ADRDA) criteria and had Braak neurofibrillary tangle (NFT) stage ≥ 4.0. Control subjects, either from the Mayo Clinic Brain Bank or the Banner Sun Health Institute, had Braak NFT stage of 3.0 or less, Consortium to Establish a Registry for Alzheimer’s Disease (CERAD) neuritic and cortical plaque densities of 0 (none) or 1 (sparse) and lacked neuropathologic diagnoses for neurodegenerative diseases. Temporal cortex samples underwent RNA extractions via the Trizol/chloroform/ethanol method, followed by DNase and clean-up of RNA using Qiagen RNeasy Mini Kit and Qiagen RNase-Free DNase Set. The quantity and quality of RNA samples were determined by the Agilent 2100 Bioanalyzer using the Agilent RNA 6000 Nano Chip. Samples included in this study all have RIN ≥ 5.0. After quality control procedures, final samples included in this study consisted of 80 Alzheimer’s disease patients and 69 controls. In a subset of 38 Alzheimer’s disease samples (18 TDP-43 positive and 20 negative) the presence of TDP-43 inclusion bodies was determined by immunohistochemistry with antibodies directed against pathological TDP-43 [5].

RNA sequencing Library preparation and sequencing of the samples were conducted at the Mayo Clinic Genome Analysis Core using TruSeq RNA Sample Prep Kit (Illumina, San Diego, CA). The library was sequenced on Illumina HiSeq2000 instruments, generating 101 base-pair, paired-end raw reads. Raw reads were processed through MAPR-Seq pipeline v1.0 [39]. Reads of low base-calling Phred scores were removed, the remaining reads were aligned to the human reference genome build hg19 using Tophat v2.0.12 [91], and were counted for genes using Subread 1.4.4 [51]. Quality control measures were obtained from both pre-alignment and post-alignment reads using RSeQC toolkit and fastQC (https://www.bioinformatics.babraham.ac.uk/projects/fastqc/). Samples that had high RNA degradation, low reads mappability, or inconsistency between recorded sex and estimated sex using RNA-seq chromosome Y expression were removed from downstream analysis. Additional information is also available in our previous publications [2, 3, 98].

The Mount Sinai Brain Bank (MSBB) RNA-seq data was downloaded from the Accelerating Medicines Partnership–Alzheimer’s Disease (AMP–AD) Knowledge Portal (syn3157743) as previously described [97]. For the MSBB RNA-seq data, pre-mortem clinical diagnosis was used to categorize Alzheimer’s disease patients and controls. We used trimmed mean of M-values (TMM) provided by the MSBB study which were already adjusted for batch, sex, race, age at death, PMI, RNA integrity number, exonic rate and ribosomal RNA rate.

For both RNA-seq datasets, reads of full-length *STMN2* (chr8: 80,523,049–80,578,410 in hg19), *UNC13A* (chr19: 17,712,145–17,799,163 in hg19), the cryptic exon of *STMN2* (chr8: 80,529,057–80,529,284 in hg19) and the cryptic exon of *UNC13A* (chr19: 17,753,223–17,753,350 in hg19) were counted for each sample using the bedtools program (v2.27.1) [77] with at least 75% overlap in the region of interest. These counts were then normalized by the total number of reads of all genes (counts per million reads, CPM), followed by Log2 transformation to obtain the expression levels in Log2(CPM). Plots were created using the ggplot2 package in R and p-values were obtained using the Wilcoxon rank-sum test. Chi-Square tests were performed using the chisq.test function and the plots were created using the ggplots package (v3.36) in R. In the plot of stratified Braak for the Mayo Clinic cohort, non-integer scores (*0.5) were rounded to the previous integer, leading to 69, 6, 34 and 40 subjects with scores 0–III, IV, V and VI, respectively. The Braak stages 0 to III were considered controls and pooled together for the analysis. Information about the presence or absence of TDP-43 was available only for 38 cases, most of them with Braak stages of V (4 TDP-43 positive and 9 negative) or VI (13 TDP-43 positive and 10 negative).

### Data visualization of iCLIP data

Individual-nucleotide resolution cross linking and immunoprecipitation (iCLIP) for TDP-43 in SH-SY5Y cells (ERR039854) previously generated by Tollervey et al. [90] was mapped to the human genome GRCh38/hg38 using star/2.7.9a. Sorted bam files were visualized and plotted using sashimi plot function in IGV (version 2.12.3) for the respective genes of interest (*UNC13A* and *STMN2*).

### Statistical analysis

Statistical analysis was performed using GraphPad Prism 8.0 software. Normality was tested using D’Agostino & Pearson test. When all groups were normally distributed, differences between groups were tested using an unpaired t-tests (for comparison of two groups) or one-way ANOVA, followed by Holm-Šídák’s Multiple comparisons post-hoc test (for comparison of more than two groups). When at least one group was not normally distributed, differences between groups were tested using Mann–Whitney test (for comparison of two groups) or Kruskal–Wallis, followed by a Dunn’s Multiple comparisons post-hoc test (for comparison of more than two groups). Correlations were tested using the Spearman’s rank correlation coefficient. A p-value of ≤ 0.05 was considered statistically significant. The following code was used to indicate the level of significance: * ≤ 0.05; ** ≤ 0.01; *** ≤ 0.001.

### Data availability

The data from this study are available from the corresponding authors, upon request. Mayo Clinic brain RNA-seq data are available from the AD Knowledge Portal (syn5550404). MSBB RNA-seq data are available from the AD Knowledge Portal (syn3157743).

## Results

### Different sensitivity of *STMN2* and *UNC13A* RNAs to TDP-43 loss

**Fig. 1:**
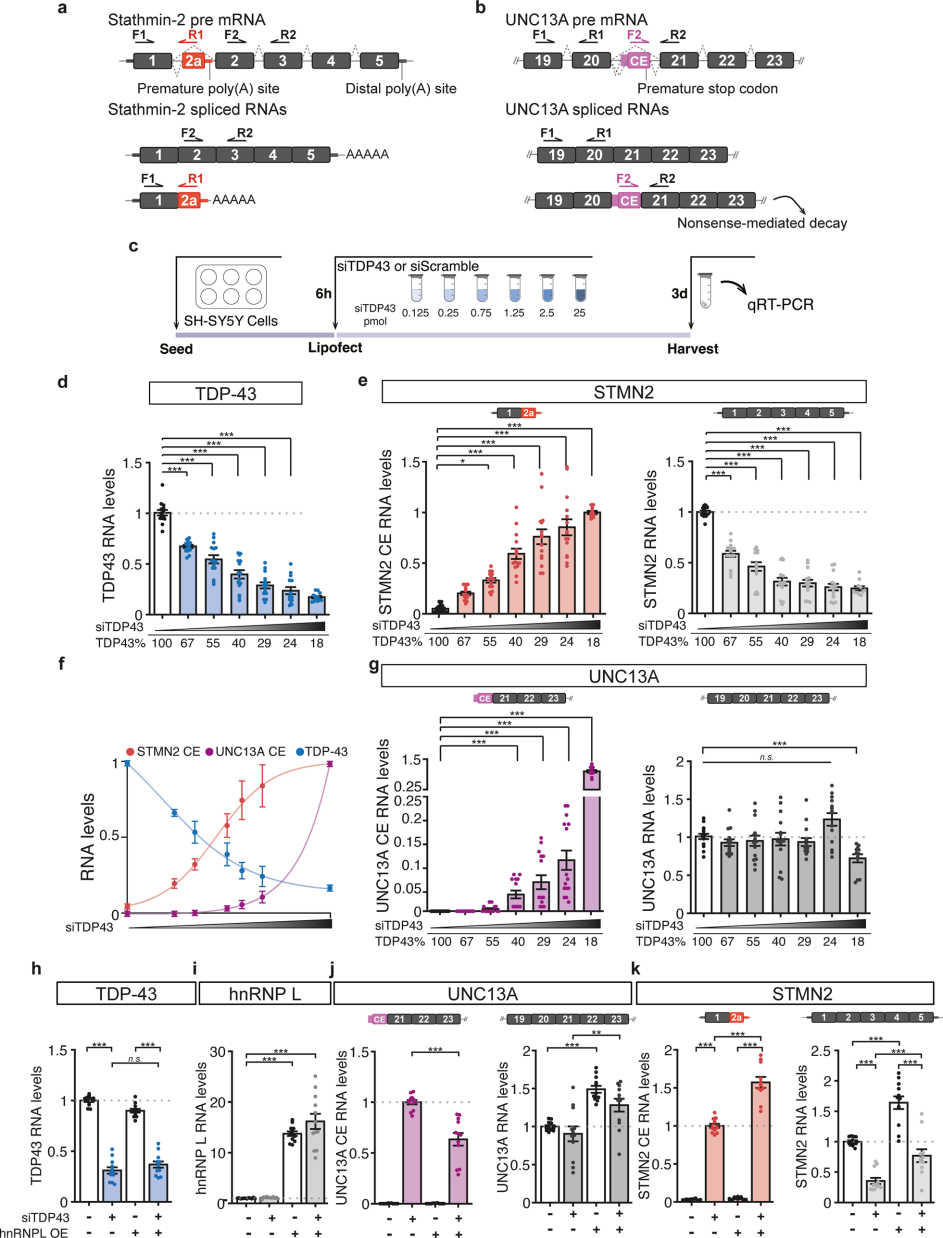
Impact of reduced TDP-43 levels on the processing of *STMN2* and *UNC13A* mRNAs. **a**, **b** Schemes representing *STMN2* (**a**) and *UNC13A* (**b**) constitutive exons (black) and cryptic exons (CE, red and purple, respectively) that are included upon TDP-43 loss of function. Forward (F) and reverse (R) primers’ location used to quantify the different transcripts by qRT-PCR in **e**–**g**, **j**, **k** are depicted. **c** Scheme of experimental design to test the sensitivity of *UNC13A* and *STMN2* cryptic exons to TDP-43 knockdown in neuroblastoma (SH-SY5Y) cells treated with increasing amounts of siRNA against TDP-43 (siTDP43) for 3 days. **d**–**g** qRT-PCR quantification of the RNA levels of *TDP-43* (**d**, blue), *STMN2* transcripts with cryptic exon (**e**, red), *STMN2* full-length (**e**, light gray), *UNC13A* transcripts with cryptic exon (**g**, purple) and *UNC13A* full-length (**g**, dark gray). As control, cells were treated with a scramble siRNA. **f** Levels of *TDP-43* (blue), *STMN2* cryptic exon (red) and *UNC13A* cryptic exon (purple) in response to TDP-43 knockdown. Data were fitted using a non-linear curve-fit in Prism, and the x axis is plotted with a log10 scale. **h**–**k** SH-SY5Y cells genetically modified to overexpress hnRNP L or a GFP control and treated with siRNA against TDP-43 for 3 days (2.5 pmol). qRT-PCR was used to determine the expression levels of *TDP-43* (blue, **h**), *hnRNP L* (**i**, gray), *UNC13A* transcripts with (**j**, purple) and without cryptic exon (**j**, dark gray) and *STMN2* transcripts with (**k**, red) and without cryptic exon (**k**, light gray). **d**–**k** RNA levels were normalized to *GAPDH* and to the control group. Levels of full-length RNAs were normalized to cells treated with the control siRNA and transcripts with cryptic exons were normalized to cells treated with highest dose of TDP-43 siRNA. Results from 4 to 5 independent experiments were plotted with each dot representing a technical replicate and bars representing mean ± SEM. Normal distribution of data was tested using D’Agostino & Pearson test and one-way ANOVA, followed by Holm-Šídák’s Multiple comparisons post-hoc test (parametric) or Kruskal–Wallis, followed by a Dunn’s Multiple comparisons post-hoc test (non-parametric) were performed accordingly

Both *STMN2* and *UNC13A* RNAs are mis-spliced upon reduction of nuclear TDP-43 with incorporation of cryptic exons to their mRNAs, resulting in decreased levels of full-length transcripts (Fig. 1a,b) [16, 42, 57, 62]. Titration of TDP-43 suppression via a small-interfering RNA (siRNA) dose response achieved a nearly continuous range of TDP-43 expression from 100 to 18% of the level in cells treated with a control siRNA (Fig. 1c,d). This approach enabled direct comparison of relative rates of cryptic exon incorporation into *UNC13A* and *STMN2* transcripts and revealed that *STMN2* pre-mRNA is more sensitive to TDP-43 disruption than *UNC13A* (Fig. 1e–g). Indeed, misprocessing of *STMN2* RNA and significant reduction of full-length *STMN2* were detected following only 33% reduction of TDP-43 level (Fig. 1d–f; 0.125 pmol siRNA leading to 67% of normal TDP-43 levels). In contrast, *UNC13A* cryptic exon was only detected following 60% reduction of TDP-43 and was associated with significant loss of full-length *UNC13A* only after 80% of TDP-43 loss (Fig. 1d,f,g; 0.75 and 25 pmol of siRNA leading to 40% and 18% of normal TDP-43 levels, respectively). Five days after treating cells with the highest dose of siRNA (25 pmol), 70% reduction of TDP-43 led to 60% reduction in full length *STMN2* but only 30% reduction in *UNC13A* (Sup. Fig. S1). Notably, treatment with 25 pmol of siTDP-43 was not associated with cellular toxicity (Sup. Fig S2a). Consistently, we detected misprocessing of *STMN2* (Sup. Fig. S2b) but not *UNC13A* (Sup. Fig. S2c) in neuroblastoma SH-SY5Y cells homozygous for a N352S mutation in *TARDBP* which results in a decrease of TDP-43 mRNA level to around 60% of wild-type cells (Sup. Fig. S2d) [62]. Analysis of previously published RNA sequencing datasets [44, 55, 62] corroborated these results (Sup. Fig. S3). Indeed, SH-SY5Y cells with TDP-43 downregulation to 25% of control cells [62], yielded a 85% reduction of full-length *STMN2*; whereas, full-length *UNC13A* was only reduced by 20% (Sup. Fig. S3a). Notably, this relative resistance of *UNC13A* to TDP-43 loss occurs in SH-SY5Y cells while sequencing of this line revealed homozygosity for the ALS-associated risk allele (G) at the SNP rs12973192 that leads to an increased likelihood of *UNC13A* cryptic exon incorporation [16, 57]. In addition, laser-microdissected motor neurons of sporadic ALS patients [44] have significantly reduced full-length *STMN2* transcripts [62] while *UNC13A* RNA levels were not significantly impacted (Sup. Fig. S3b). Finally, while both *STMN2* and *UNC13A* cryptic exons were detected in FAC sorted cortical neurons lacking nuclear TDP-43 [16, 55, 57], only full-length *STMN2* transcripts were significantly reduced (Sup. Fig. S3c, left panel) while *UNC13A* RNA levels were not significantly impacted (Sup. Fig. S3c, right panel). Collectively, these results indicate that while *STMN2* and *UNC13A* pre-mRNAs are both misprocessed upon TDP-43 loss, *STMN2* is more sensitive to mild TDP-43 disruption than *UNC13A*.

Several factors may underly the different sensitivities of *STMN2* and *UNC13A* to TDP-43 loss-of-function including the binding patterns of TDP-43 on the pre-mRNAs and the level of expression of both transcripts. Analysis of a previously published individual-nucleotide resolution cross linking and immunoprecipitation (iCLIP) dataset in SH-SY5Y cells [90] identified a specific binding of TDP-43 on *STMN2* cryptic exon while no read mapped in the region surrounding the *UNC13A* cryptic exon (Sup. Fig. S4a,b). This result may be due to the low level of *UNC13A* expression compared to *STMN2* in SH-SY5Y cells (200 *STMN2* transcripts per million compared to 20 *UNC13A* transcripts per million in control SH-SY5Y cells, Sup. Fig. S3a). Notably, iCLIP from SH-SY5Y cells overexpressing *UNC13A* minigenes demonstrated TDP-43 binding on a large region of intron 20 spanning approximately 800 bp [16] (Fig. S5a). Consistently, the region bound by TDP-43 is highly UG-rich with not only UG repeats but also motifs interrupted by adenines that were previously identified to be bound by TDP-43 [16, 74, 90]. This binding pattern strikingly contrasts with the discreet binding site of TDP-43 on *STMN2* that spans only 24 nucleotides [10, 62] (Fig. S5b), likely contributing to the high sensitivity of *STMN2* transcripts to TDP-43 loss.

It is also possible that other RNA binding proteins influence TDP-43-dependent cryptic splicing events. Indeed, overexpression of the heterogeneous nuclear ribonucleoprotein L (hnRNP L) was recently shown to mitigate the incorporation of cryptic exon in *UNC13A* upon TDP-43 loss [43]. We used genetically modified SH-SY5Y cells over-expressing either hnRNP L or a GFP control to determine whether hnRNP L also represses the incorporation of *STMN2* cryptic exon upon siRNA-mediated reduction of TDP-43 (Fig. 1h). hnRNP L expression was not altered by TDP-43 loss (Fig. 1i), but as previously reported [43], significantly repressed the aberrant incorporation of *UNC13A* cryptic exon and increased the level of full length *UNC13A* (Fig. 1j). In contrast, both full length and cryptic exon-containing *STMN2* transcripts were increased by overexpression of hnRNP L (Fig. 1k).

### *STMN2* and *UNC13A* RNAs are mis-processed in Alzheimer’s disease patients with TDP-43 proteinopathy

**Fig. 2:**
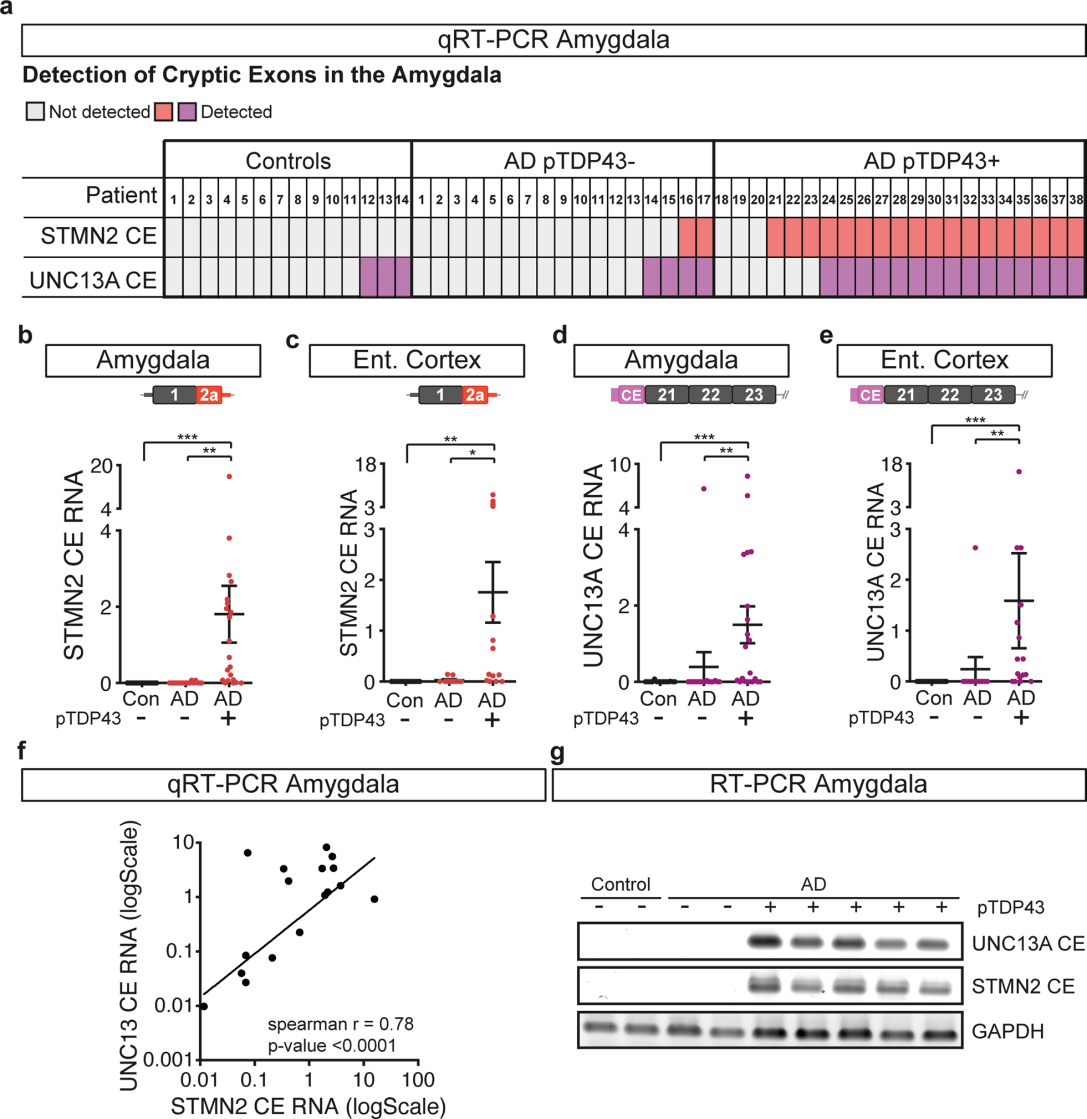
*STMN2* and *UNC13A* RNAs are mis-processed in post-mortem brain of approximately half of patients with Alzheimer’s disease. **a** Table depicting the detection of *STMN2* (red) or *UNC13A* (purple) cryptic exons in the amygdala of controls and Alzheimer’s disease patients with (+) and without (−) phosphorylated TDP-43 pathology. **b**–**e** Levels of truncated *STMN2* RNA (red) or *UNC13A* cryptic exon (purple) measured by qRT-PCR in post-mortem amygdala (**b**, **d**) or entorhinal cortex (**c**, **e**) from patients with Alzheimer’s disease (AD) associated ( +) or not (−) with accumulation of phosphorylated TDP-43. Normal distribution of data was tested using D’Agostino & Pearson test and a one-way ANOVA followed by a Sidak’s multiple comparisons post-hoc test (parametric), or a Kruskal–Wallis, followed by a Dunn’s Multiple comparisons post-hoc test (non-parametric) were performed accordingly. **f** Log–log plot of *STMN2* and *UNC13A* cryptic exon levels detected in the amygdala of Alzheimer’s disease patients. Correlation was determined using spearman rank coefficient and curve fitting with non-linear regression. **g** RT-PCR confirming the detection of *STMN2* and *UNC13A* cryptic exons in a subset of the patients with Alzheimer’s disease associated with phosphorylated TDP-43 (pTDP-43) pathology

Aberrant accumulations of TDP-43 are found in approximately half of patients with Alzheimer’s disease, with the amygdala and entorhinal cortex, two anatomically adjacent regions, being the most frequently affected areas [5, 34]. To test whether TDP-43 pathology in Alzheimer’s disease patients leads to RNA mis-processing of *STMN2* and *UNC13A*, we performed qRT-PCR using RNA from the amygdala and entorhinal cortex of 38 Alzheimer’s disease patients and 14 age-matched control individuals without significant confounding neuropathology (Sup. Table 2). Abnormal *STMN2* transcripts were detected in the amygdala of 20 out of 38 patients and in the entorhinal cortex of 13 out of 27 patients with Alzheimer’s disease, and none of the control individuals (Sup. Fig. S6a–c and Sup. Table 3). We also tested misprocessing of *UNC13A* RNA in these areas and detected *UNC13A* cryptic exon in 19/38 amygdala samples and 13/27 entorhinal cortex samples from Alzheimer’s disease patients and at very low levels (qRT-PCR cycle thresholds Cts above 35 cycles) in the amygdala of 3 control individuals (Sup. Fig. S6d–f and Sup. Table 3). We then performed immunostaining to test the presence of hyperphosphorylated TDP-43 aggregates in the amygdala of these patients, and determined that the detection of *STMN2* and *UNC13A* cryptic exons correlated with the presence of TDP-43 pathology (Fig. 2a–e and Sup. Table 3). Indeed, *STMN2* cryptic exon was detected in the amygdala of 18 out of 21 Alzheimer’s disease patients positive for phosphorylated TDP-43 and was detected at low levels (Cts above 37; Sup. Fig. S6f) in only 2 out of 17 patients without detectable phosphorylated TDP-43 aggregates (Fig. 2a,b and Sup. Table 3). *UNC13A* cryptic exon was identified in the amygdala of 15 out of 21 Alzheimer’s disease patients with phosphorylated TDP-43 aggregates and only 4 patients without detected aggregates (Fig. 2a,d and Sup. Table 3). Similar results were obtained in the entorhinal cortex (Fig. 2c,e and Sup. Table 3). Notably, we observed a correlation between the levels of *STMN2* and *UNC13A* cryptic exons detected in the amygdala of Alzheimer’s disease patients (Fig. 2f, Spearman r = 0.78, p < 0.0001), strengthening the link between detection of cryptic exons and loss of TDP-43 function. Finally, our observations were confirmed by RT-PCR showing the amplification of cryptic exons of both *STMN2* and *UNC13A* transcripts in the amygdala of Alzheimer’s disease patients with TDP-43 pathology, but not in the controls or Alzheimer’s disease patients without TDP-43 pathology (Fig. 2g).

TDP-43 pathology follows a stereotypic spread from amygdala to other brain areas as Alzheimer’s disease progress [34, 67]. We classified postmortem tissues according to the presence of TDP-43 proteinopathy in the amygdala (stage I), hippocampus (stage II) and frontal cortex (stage III) following recent recommendations [67]. Staging was possible in 14 TDP-43 positive patients for which tissues were available from all three brain regions (Sup. Table 3). Only 4 out of 14 patients displayed phosphorylated TDP-43 aggregates in the frontal cortex (stage 3). In these cases, only a scarce number of inclusions were observed in the frontal cortex compared to amygdala and entorhinal cortex (Sup. Fig. S7). Consistently, *STMN2* cryptic exon was only rarely detected in frontal cortex (1/19), occipital cortex (1/19) and cerebellum (4/19) in Alzheimer’s disease patients (Sup. Fig. S6a and Sup. Table 3). Notably, all the patients with cryptic exons detected in frontal cortex, occipital cortex and cerebellum also had cryptic exons in the amygdala (Sup. Table 3).

### Detection of *STMN2* and *UNC13A* cryptic exons in the amygdala correlates with the burden of phosphorylated TDP-43 but not tau or amyloid-β

**Fig. 3:**
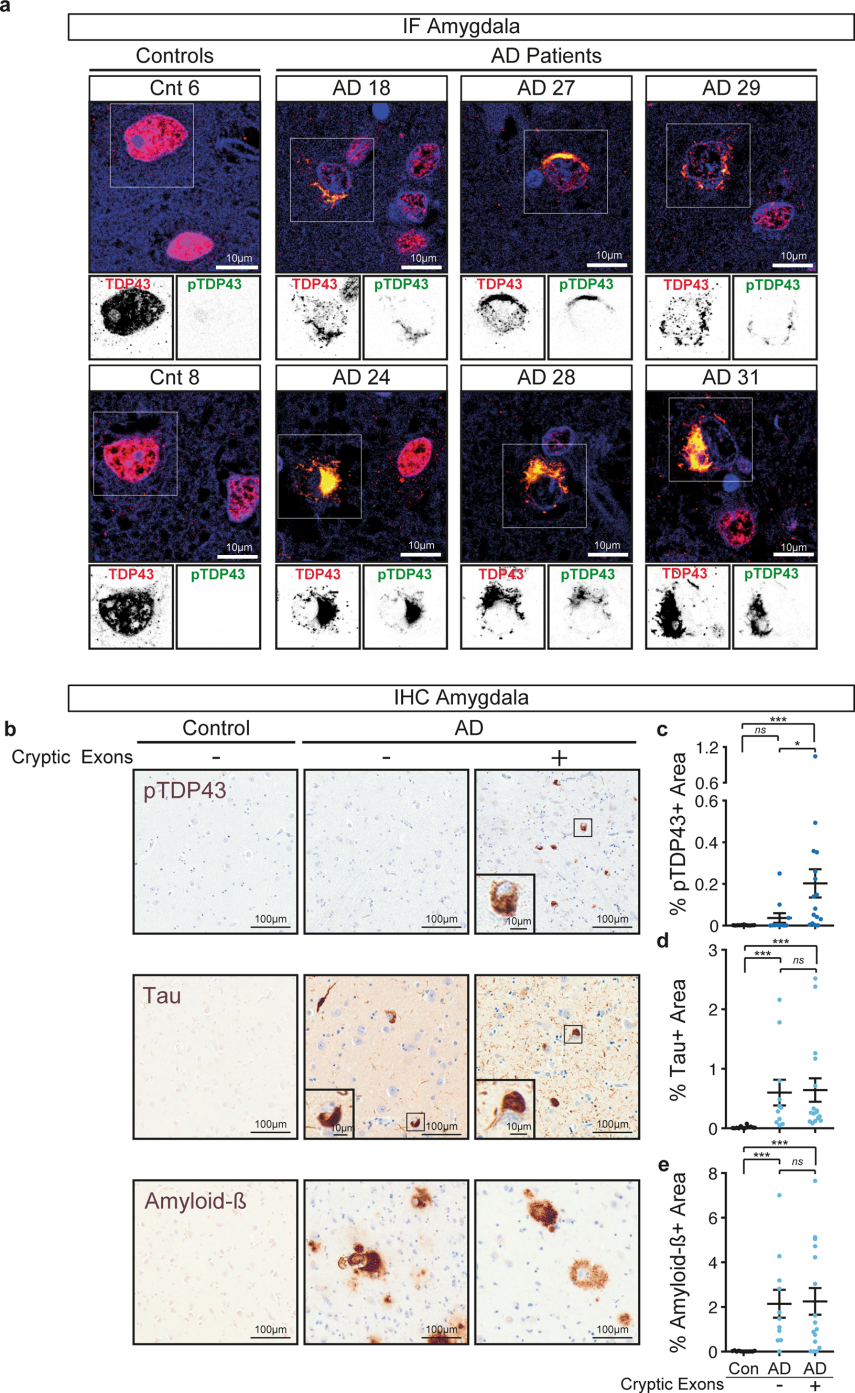
Detection of cryptic exons in the amygdala from Alzheimer’s disease patients correlates with the burden of phosphorylated TDP-43 but not Tau or Amyloid-β. **a** Representative immunofluorescence images showing full-length TDP-43 (red), phosphorylated TDP-43 (green) and DAPI (blue) of two controls and six Alzheimer’s disease patients. Top panels show merged images and lower insets show grayscale images of TDP-43 or phosphorylated TDP-43. Scale bar is 10 µm. **b** Example micrographs of post-mortem brain tissue from one control and two Alzheimer’s disease patients after immunohistochemical detection of phosphorylated TDP-43 (pTDP43), total Tau and Amyloid-β. Scale bar is 100 µm in larger panels, and 10 µm in insets. **c**–**e** Percentage of area occupied by phosphorylated TDP-43 (**c**), tau (**d**) or amyloid-β (**e**) staining in the amygdala of controls or Alzheimer’s disease patients with or without detection of cryptic exons. For each case five regions of interest of equal size were selected and the positive signal for the different markers determined and averaged. Data represent mean ± SEM, each dot represents an individual patient. Normal distribution of data was tested using D’Agostino & Pearson test and one-way ANOVA, followed by Holm-Šídák’s Multiple comparisons post-hoc test (parametric) or Kruskal–Wallis, followed by a Dunn’s Multiple comparisons post-hoc test (non-parametric) were performed accordingly

Since Alzheimer’s disease neuropathology is characterized by the accumulation of the two canonical proteins, amyloid-β and tau [14], we used semi-automated quantification to determine the area occupied by pathological inclusions of phosphorylated TDP-43, tau and amyloid-β in post-mortem amygdala of Alzheimer’s disease patients with (CE +) or without (CE−) *STMN2* or *UNC13A* cryptic exons (Fig. 3). TDP-43 pathology is characterized by both cytoplasmic inclusions and loss of nuclear TDP-43 that appears to be an early pathogenic mechanism in TDP-43 proteinopathies [64, 68, 96]. Immunofluorescence staining confirmed that cells containing phosphorylated TDP-43 cytoplasmic inclusions did have nuclear clearance of TDP-43 consistent with TDP43 loss of function in regulating splicing, while neighbouring cells without inclusions had normal nuclear TDP-43 (Fig. 3a). Quantification of TDP-43 nuclear loss is challenging compared to quantification of phosphorylated TDP-43 aggregates [5, 7, 13, 21, 29, 34, 35, 54, 92], hence we have correlated the presence of cryptic exons with the burden of phopho-TDP-43 pathology (Fig. 3b,c). As expected, there were higher levels of phosphorylated TDP-43, tau and amyloid-β accumulations in Alzheimer’s disease patients compared to control individuals (Fig. 3b–e). However, while the pathological burden in tau (Fig. 3d) or amyloid-β (Fig. 3e) were not significantly different in tissues with or without cryptic exons, we observed higher pathological burden of TDP-43 in patients with accumulation of *STMN2* or *UNC13A* cryptic exons (Fig. 3c). Notably, the burden of TDP-43 pathology (percentage of area positive for TDP-43) positively correlated with the levels of both *STMN2* (Spearman r = 0.52; p-value = 0.006) and *UNC13A* (Spearman r = 0.56; p-value = 0.002) cryptic exons in the amygdala. Collectively these results demonstrate that mis-processing of *STMN2* and *UNC13A* RNAs is a pathological hallmark of TDP-43-associated Alzheimer’s disease, with amygdala and entorhinal cortex being the most affected areas.

### RNA-seq identifies *STMN2* and *UNC13A* disruption in a subset of Alzheimer’s disease patients

**Fig. 4:**
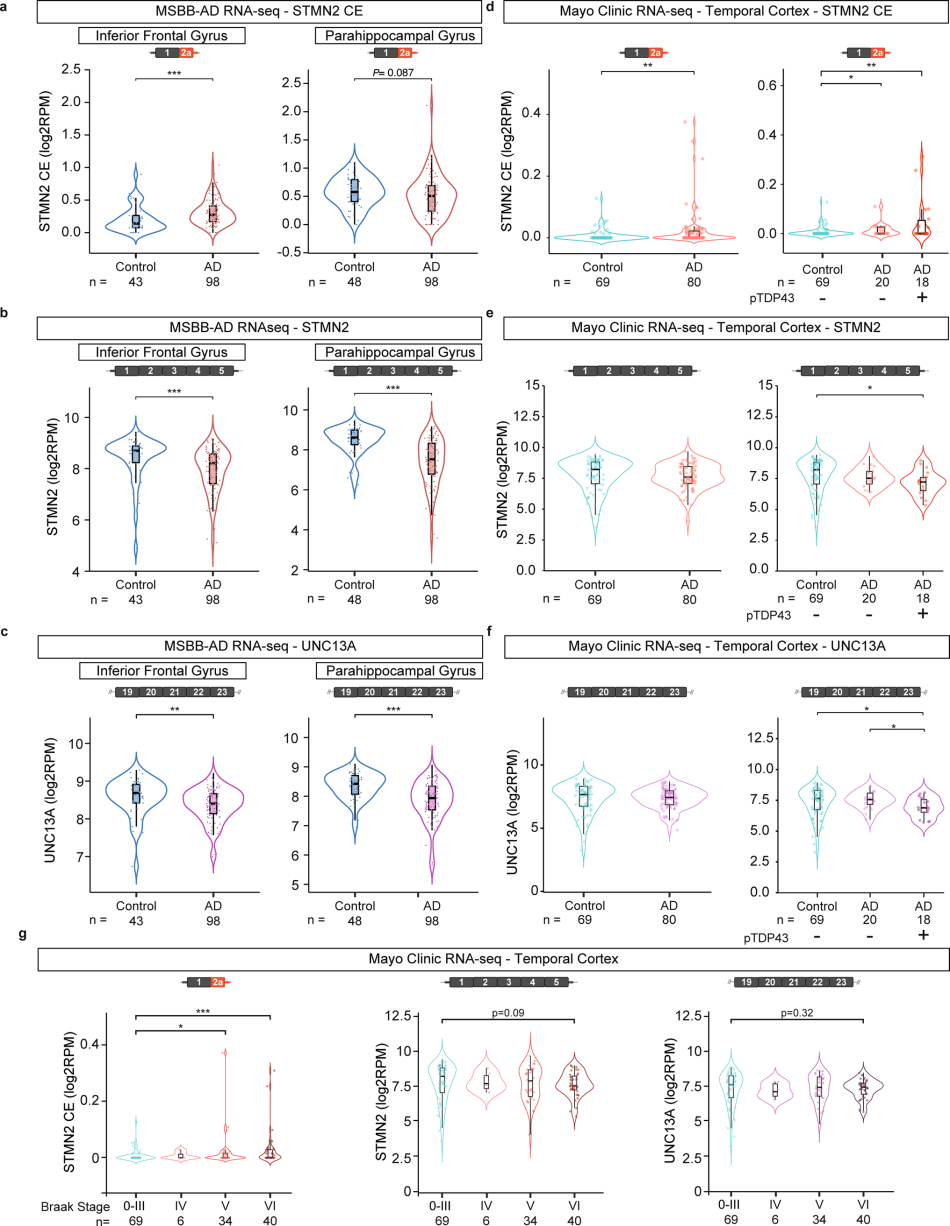
Analysis of *STMN2* and *UNC13A* mRNAs in RNA-seq datasets from Alzheimer’s disease post-mortem tissues. Expression of *STMN2* and *UNC13A* was analyzed using published RNA-seq datasets from the Mount Sinai/JJ Peters VA Medical Center Brain Bank (MSBB-AD) [97] (**a**–**c**) and from the Mayo Clinic Brain Bank [2, 3, 98] (**d**–**g**). Levels of *STMN2* cryptic exon (red, **a**, **d**), *STMN2* full-length transcript (red, **b**, **e**) and *UNC13A* full-length transcript expression (purple, **c**, **f**) were determined in the parahippocampal and inferior frontal gyri (**a**–**c**) or temporal cortex (**d**–**g**) of Alzheimer’s disease patients compared to controls. **g** Levels of *STMN2* cryptic exon (left, red), *STMN2* full-length (middle, red) and *UNC13A* full-length (right, purple) in post-mortem brain tissue from controls (Braak stage 0–III) and Alzheimer’s disease patients segregated according to their Braak stage (Braak stages IV–VI)

We analyzed *STMN2* and *UNC13A* transcripts in bulk RNA-seq from post-mortem brain regions of control and Alzheimer’s disease patients from data available through the Mount Sinai/JJ Peters VA Medical Center Brain Bank (MSBB-AD) [97] and an independent dataset generated at Mayo Clinic [2, 3, 98]. In the Mount Sinai dataset, RNA-seq of the parahippocampal gyrus, inferior frontal gyrus, frontal pole, and superior temporal gyrus from more than 40 control individuals and 95 Alzheimer’s disease patients were analyzed (Fig. 4a–c, Sup. Figs. S8, S9). Of note, no information was available concerning the presence or absence of TDP-43 pathology in this cohort. However, we observed a significant increase of truncated *STMN2* in the inferior frontal gyrus (Fig. 4a) of Alzheimer’s disease patients; whereas, full-length *STMN2* RNA levels were significantly decreased in Alzheimer’s disease patients compared to controls, in all the regions tested (Fig. 4b and Sup. Fig. S8b). While the number of reads mapping to *UNC13A* cryptic exon was extremely low in all samples, a significant decrease in *UNC13A* full-length was detected in the inferior frontal gyrus, parahippocampal gyrus and superior temporal gyrus from Alzheimer’s disease patients compared to control individuals (Fig. 4c and Sup. Fig. S8c). The Mayo Clinic dataset [2, 3, 98] consisted of bulk RNA-seq of the temporal cortex (superior temporal gyrus) from 69 control individuals and 80 Alzheimer’s disease patients. Here, TDP-43 pathology was assayed in 38 of the Alzheimer’s disease patients with 18 showing accumulation of phosphorylated TDP-43, consistent with previous reports of approximately 50% of Alzheimer’s disease patients having TDP-43 pathology [5, 7, 13, 21, 29, 34, 35, 54, 92]. Analysis of the Mayo Clinic dataset showed a significant enrichment of *STMN2* cryptic exon in the temporal cortex of Alzheimer’s disease patients compared to controls (Fig. 4d) and a significant decrease in full-length *STMN2* only in Alzheimer’s disease patients with TDP-43 pathology compared to controls (Fig. 4e). *UNC13A* full-length was significantly decreased in patients with TDP-43 pathology (Fig. 4f). We further explored this dataset by subdividing Alzheimer’s disease cases (including cases with and without TDP-43 pathology) according to their Braak stages, a method to classify the evolution of the disease by evaluating the spread of tau pathology in Alzheimer’s disease brain [14, 15]. This analysis revealed a significant increase of *STMN2* cryptic exon in the more advanced Braak stages (Braak stages V and VI), a trend for decrease in full-length *STMN2* in the Mayo Clinic dataset, and a significant decrease in the full-length transcripts of *STMN2* and *UNC13A* in the Mount Sinai dataset (Fig. 4g and Sup. Fig. S9). In the cases from Mayo Clinic with Braak stages V and VI for which the TDP-43 status was available, we observed a significant increase of *STMN2* cryptic exon in the temporal cortex of patients with Braak stage VI and TDP-43 pathology (Sup. Fig. 10a). Full-length *STMN2* and *UNC13A* transcripts were also significantly reduced in both Braak stages V and VI when comparing brains with and without phosphorylated TDP-43 (Sup. Fig. 10).

Collectively this analysis establishes, in a large sample population, that *STMN2* and *UNC13A* transcripts are altered in Alzheimer’s disease patients with advanced Braak stages when the burden of TDP-43 pathology is high.

## Discussion

Alzheimer’s disease is a heterogenous disease, and precision medicine approaches to fine-tune diagnosis and treatment offer real promise for patients. TDP-43 pathology co-occurs with Alzheimer’s disease in a subset of patients, which is also termed Alzheimer’s disease with LATE-NC, likely contributing to neural system dysfunction in those patients. Indeed, TDP-43 pathology in Alzheimer’s disease is associated with more severe cognitive decline and neurodegeneration [17, 18, 32–34, 36, 37, 99], underscoring the need to stratify Alzheimer’s disease patients according to their TDP-43 pathology status. In addition, the higher likelihood of TDP-43 lesions in patients with typical Alzheimer’s disease pathology compared to the general population raises the question of whether there are synergistic or underlying genetic or environmental factors that contribute to both Alzheimer’s disease and TDP-43 pathology, but this is currently unknown. Notably, TDP-43 pathology has also been described in limbic regions of older patients with and without dementia (LATE-NC) who lack tau and amyloid-β pathologies [66, 67, 81]. Our results support the use of TDP-43-dependent splicing alterations to sub-categorize patients with respect to TDP-43 dysfunction. RNA-seq has been used before to demonstrate disease-specific splicing alterations in Alzheimer’s disease [9, 27, 78, 94]. Recent reports have detected cryptic exons by using qRT-PCR or RT-PCR in post-mortem tissues from patients with Alzheimer’s disease [22, 87]. Our study extends on those findings by demonstrating that *STMN2* and *UNC13A* disruption from TDP-43 loss-of-function is detected in large RNA-seq datasets and can distinguish between Alzheimer’s disease patients with and without TDP-43 pathology. Notably, previous studies observed a non-significant tendency for decreased levels of full-length *STMN2* RNA in the temporal cortex and hippocampus of a subset of patients with Alzheimer’s disease [73], as well as a decrease in *STMN2* RNA in inhibitory neurons and oligodendrocytes of Alzheimer’s disease patients [60]. Although these studies did not correlate the transcriptional changes to TDP-43 pathology, they independently confirm *STMN2* as a relevant mechanistic disease target. Importantly, we now demonstrate that cryptic exon detection correlates with TDP-43 burden, but not with amyloid-β and tau in the amygdala of Alzheimer’s disease patients. TDP-43 pathology follows a stereotypic spread as Alzheimer’s disease progress [34], and we observe that *STMN2* and *UNC13A* are indeed significantly disrupted in temporal cortex of patients with high Braak stages. The *STMN2* cryptic exon was detected in most of the cases with phosphorylated TD-43 aggregates except for 3 out of 21 tissues tested. Although we cannot exclude that additional protective factors (genetic or environmental) may prevent mis-processing of RNA targets in a subset of patients, it is noteworthy that, in a given brain region, TDP-43 pathology is spatially heterogeneous and expression of cryptic exons may be below the detection limit in the assessed tissue sample. Inversely, *STMN2* cryptic exon was detected in 2 out of 17 brains without apparent phosphorylated TDP-43 aggregates which is consistent with reports of TDP-43 nuclear loss without cytoplasmic aggregates in a subset of patients with FTD or Alzheimer’s disease [64, 87].

Our systematic analysis indicates that *STMN2* is more sensitive than *UNC13A* to decrease in TDP-43 levels; whereas, a more robust TDP-43 suppression is necessary to observe the dose response of *UNC13A* cryptic exon generation. This is consistent with previous observation that depletion of at least 50% of TDP-43 encoding RNA was required to induce *UNC13A* RNA mis-splicing [16]; whereas, *STMN2* mis-processing is triggered by less than 30% reduction of TDP-43 [62]. In addition, cryptic exons are more commonly identified in *STMN2* than *UNC13A* within post-mortem tissues from patients with either ALS and/or FTD [16, 25]. Our RNA-seq analysis also shows that the *STMN2* cryptic exon is enriched in tissues of Alzheimer’s disease; whereas, no significant enrichment of *UNC13A* cryptic exon was detected. *STMN2* expression levels are tenfold higher than *UNC13A* both in SH-SY5Y cells and human tissues, with *STMN2* being among the 25 most expressed genes in laser-microdissected human motor neurons [44, 62], which may contribute to the differential detection of *STMN2* and *UNC13A* cryptic exons. In addition, *UNC13A* cryptic exon-containing transcripts might escape detection due to a shorter half-life since they are subjected to nonsense-mediated decay while *STMN2* truncated transcripts are not [16]. However, we propose that the higher detection of *STMN2* cryptic exons is also due to an intrinsic different vulnerability of *STMN2* and *UNC13A* transcripts to TDP-43 loss of function. Indeed, the binding pattern of TDP-43 on *STMN2* and *UNC13A* pre-mRNAs is distinct, with a narrow binding site consisting of 3 ugugug motifs over 22 bp in the cryptic exon (exon 2a) of *STMN2* [10, 62]; whereas, TDP-43 binds an 800 bp-long ug-rich region in intron 20 of UNC13A [16]. In addition, hnRNP L was recently found to mitigate the impact of TDP-43 loss on *UNC13A* splicing [43]. In contrast, we show that hnRNP L does not repress the inclusion of *STMN2* cryptic exon.

Identification of *STMN2* and *UNC13A* disruption in Alzheimer’s disease has major implications: (1) they are both enriched in the brain, (2) they have crucial roles in maintaining neuronal function [8, 26, 42, 45, 56, 62, 63, 80, 84, 95] and (3) they are two prominent targets of TDP-43 for which therapeutic approaches are in development [10, 16, 42, 57, 62]. STMN2 is a tubulin-binding protein [19] important for axonal maintenance and regeneration after injury [42, 62, 63, 80, 84]. Loss of *STMN2* in vivo leads to motor and sensory behavioural deficits, as well as denervation of neuromuscular junctions [26, 45, 50, 56]. Finally, even though many RNAs are affected by TDP-43 loss-of-function [42, 49, 52, 62, 72, 74, 90], restoring STMN2 levels alone is sufficient to rescue axonal transport defects and the ability of iPSC-derived motor neurons to regrow after axotomy [10, 42, 62]. UNC13A has been extensively linked to synaptic function, and it is essential for neurotransmission [8, 95]. As such, both STMN2 and UNC13A are important components of neuronal function, and given the lack of cognitive resilience and increased brain atrophies associated with TDP-43 pathology in Alzheimer’s disease [17, 18, 32, 34, 36, 37, 99], future studies should focus on exploring how these targets may contribute to neurodegeneration in Alzheimer’s disease. Importantly, strategies to either target TDP-43 cellular mislocalization [1, 4, 11, 23, 47, 58, 70, 75, 88, 89] or to prevent cryptic splicing of specific TDP-43 targets [10] are emerging for clinical development in neurodegenerative diseases with TDP-43 proteinopathy. Indeed, the usage of ASOs chemically modified to alter pre-mRNA splicing without recruiting RNase H is an approved standard of care for spinal muscular atrophy (SMA) and has become an attractive therapeutic approach for neurodegenerative diseases [12, 24]. We recently demonstrated the ability of steric binding ASOs to prevent mis-splicing and restore stathmin-2 protein level in TDP-43 deficient neurons and mouse brain [10], providing support for restoration of STMN2 as a potential therapeutic approach in TDP-43 proteinopathies. Collectively, this further supports the use of cryptic exons to stratify Alzheimer’s disease patients to enable the development of targeted therapies. It is noteworthy that approaches are also emerging to use TDP-43-dependent splicing alterations as potential biomarkers in patients with TDP-43 proteinopathy. Indeed, while PET imaging for TDP-43 pathology is not available and detection of pathological TDP-43 in biofluids has yielded conflicting results [28, 40, 41, 46, 69, 79, 86], recent studies show the feasibility of detecting aberrant peptides generated from TDP-43-dependent cryptic splicing in CSF of patients with ALS/FTD [31, 83]. Even though there are not yet reliable biomarkers based on the detection of *STMN2* or *UNC13A* cryptic exons, development of these approaches may have ground-breaking implications for subcategorization of patients and the development of personalized therapeutics in TDP-43 proteinopathies, including Alzheimer’s disease.

### Supplementary Information

Below is the link to the electronic supplementary material.

Supplementary file1 (PDF 5207 KB)

## Abbreviations

siRNA: Small-interfering RNA
STMN2: Stathmin-2
TDP-43: TAR DNA-binding protein-43
ALS: Amyotrophic lateral sclerosis
FTD: Frontotemporal dementia
LATE: Limbic-predominant age-related TDP-43 encephalopathy
LATE-NC: LATE neuropathologic changes
ASO: Antisense oligonucleotide
hnRNP L: Heterogeneous nuclear ribonucleoprotein L
iCLIP: Individual-nucleotide resolution cross linking and immunoprecipitation
