# Supplementary material for: Cryptic splicing of stathmin-2 and UNC13A mRNAs is a pathological hallmark of TDP-43-associated Alzheimer’s disease
Source: Acta Neuropathol. 2024 Jan 4;147(1):9. doi: 10.1007/s00401-023-02655-0 (PMC10766724; doi:10.1007/s00401-023-02655-0)
Supplement: Supplementary file 1 — Supplementary file1 (PDF 5207 KB) [file 401_2023_2655_MOESM1_ESM.pdf]

Cryptic splicing of Stathmin-2 and UNC13A mRNAs is a pathological hallmark of TDP-43-associated Alzheimer's disease

Ana R. A. A. Quadros[1,2,3†], Zhaozhi Li[2†], Xue Wang[4†], I. Sandra Ndayambaje[1,2], Sandeep Aryal[1,2,3], Nandini Ramesh[1,2,3], Matthew Nolan[1,2,3], Rojashree Jayakumar[2], Yi Han[1,2], Hannah Stillman[1,2], Corey Aguilar[1,2], Hayden J. Wheeler[1,2], Theresa Connors[5], Jone Lopez-Erauskin[6], Michael W. Baughn[6], Ze'ev Melamed[6], Melinda S. Beccari[6], Laura Olmedo Martínez [1,2], Michael Canori[1,2,3], Chao-Zong Lee[1,2], Laura Moran[1,2], Isabelle Draper[7], Alan S. Kopin[7], Derek H. Oakley[5], Dennis W. Dickson[8], Don W. Cleveland[6], Bradley T. Hyman[2], Sudeshna Das[2,10], Nilüfer Ertekin-Taner[8,9,10], Clotilde Lagier-Tourenne[1,2,3,10]

[†]These authors contributed equally to this work.

Author affiliations:

1 Department of Neurology, The Sean M. Healey and AMG Center for ALS, Massachusetts General Hospital, Harvard Medical School, Boston, MA, USA

2 MassGeneral Institute for Neurodegenerative Diseases (MIND), Massachusetts General Hospital, Harvard Medical School, Boston, MA, USA

3 Broad Institute of Harvard University and MIT, Cambridge, MA, USA

4 Department of Quantitative Health Sciences, Mayo Clinic, Jacksonville, FL, USA

5 Department of Pathology, Massachusetts General Hospital, Harvard Medical School, Boston, MA, USA

6 Department of Cellular and Molecular Medicine, University of California at San Diego, La Jolla, CA, USA

7 AUTTX LLC, Wellesley, MA, USA

8 Department of Neuroscience, Mayo Clinic, Jacksonville, FL, USA

9 Department of Neurology, Mayo Clinic, Jacksonville, FL, USA

10 Correspondence to: Sudeshna Das, Nilüfer Ertekin-Taner, Clotilde Lagier-Tourenne.

Full address: Department of Neurology, Massachusetts General Hospital, Harvard Medical School, Boston, MA, USA

E-mail: SDAS5@mgh.harvard.edu ; Taner.Nilufer@mayo.edu ; clagier-tourenne@mgh.harvard.edu

Running title: Loss of STMN2 and UNC13A in Alzheimer's disease

Journal: Acta Neuropathologica

Supplementary Figures and legends

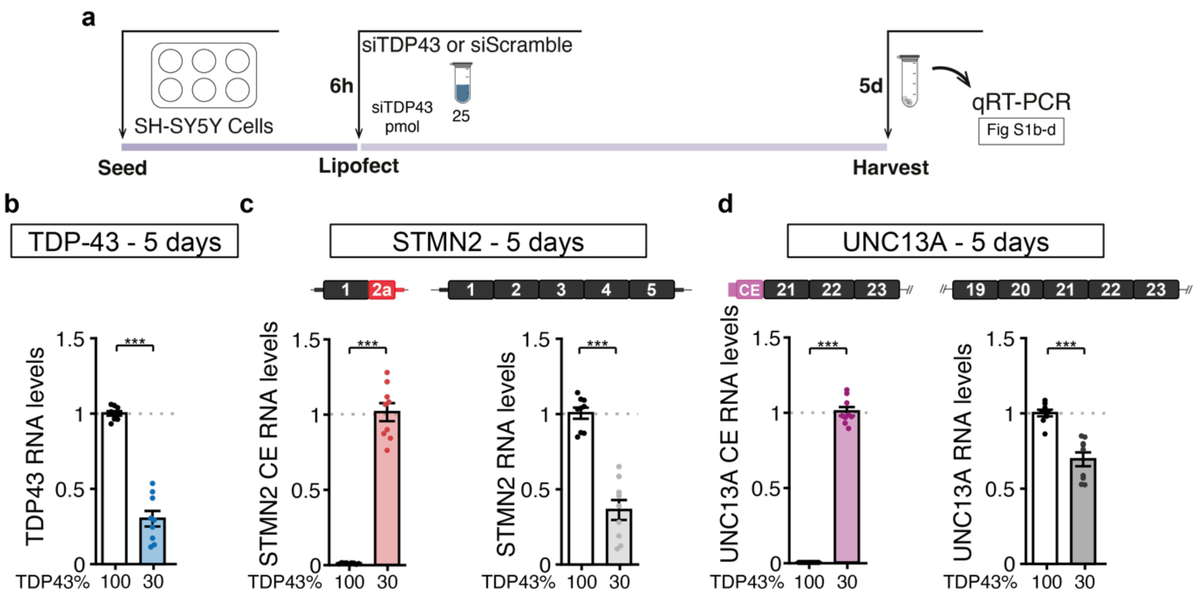

Supplementary Figure S1: Processing of *STMN2* and *UNC13A* mRNAs in neuroblastoma cells after 5 days of TDP-43 knockdown. (**a**) Scheme of experimental design for treatment of SH-SY5Y with 25 pmol of TDP-43 or scramble siRNAs during 5 days. (**b-d**) qRT-PCR was performed to quantify the RNA levels of *TDP-43* (**b**, blue), *STMN2* transcripts with cryptic exon (**c**, red), *STMN2* full-length (**c,** light gray), *UNC13A* transcripts with cryptic exon (**d**, purple) and *UNC13A* full-length (**d**, dark gray). Results from 3 independent experiments are included with each dot representing biological replicates and bars representing mean ± SEM. Normal distribution of the data was tested using D'Agostino & Pearson test and an unpaired t-test (parametric) or Mann-Whitney test (non-parametric) were performed accordingly.

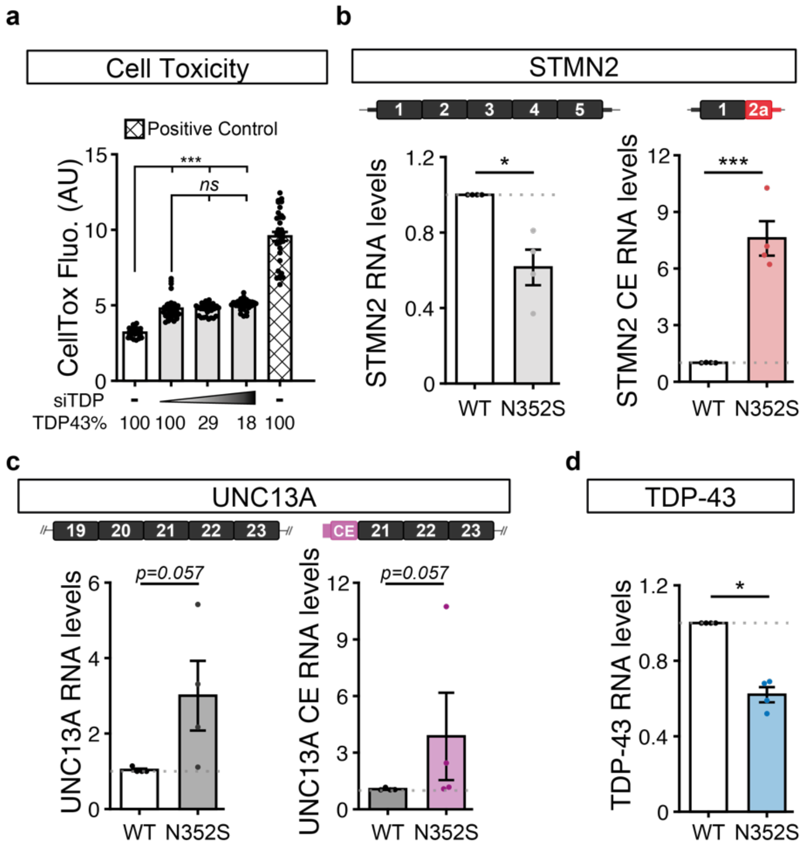

Supplementary Figure S2: Expression of *STMN2* and *UNC13A* mRNAs in neuroblastoma cells. (**a**) Potential toxicity of knocking down TDP-43 was evaluated using a CellTox green assay that measures cell-membrane integrity with a fluorescent dye, in cells treated with 2.5 pmol or 25 pmol of siTDP43 for 5 days. (**b**-**d**) RNA from SH-SY5Y cells CRISPR-edited to express an ALS-associated mutation of TDP-43 (N352S) [8] was quantified to determine the transcript levels of *STMN2* full length (**b**, light gray), *STMN2* transcripts with cryptic exon (**b**, red), *UNC13A* full length (**c**, dark gray), *UNC13A* transcripts with cryptic exon (**c**, purple) and *TDP-43* (**d**, blue). Results from 3 or 4 independent experiments are included with each dot representing replicates and bars representing mean ± SEM. Normal distribution of data was tested using D'Agostino & Pearson test and an unpaired t-test (parametric) or Mann-Whitney test (non-parametric), or a Kruskal-Wallis, followed by a Dunn's Multiple comparisons post-hoc test (non-parametric) were performed accordingly.

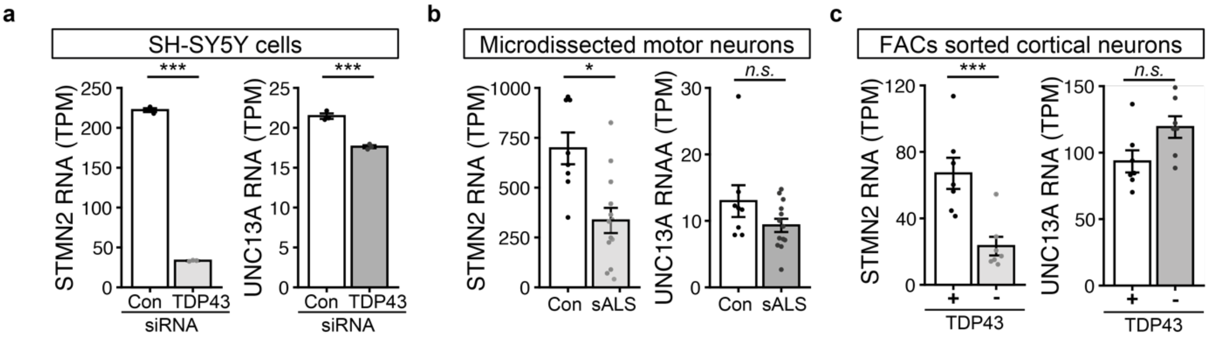

Supplementary Figure S3: Levels of *STMN2* and *UNC13A* transcripts in different human cells with disrupted TDP-43. (**a**-**c**) *STMN2* and *UNC13A* full-length levels (transcripts per million, TPM) in (**a**) SH-SY5Y neuroblastoma cells treated with siRNA against TDP-43 [8], (**b**) laser microdissected motor neurons from patients with sporadic amyotrophic lateral sclerosis (sALS) [5], and (**c**) cortical neurons FACs sorted for the presence (+) or absence (-) of nuclear TDP-43 [6]. Normal distribution of data was tested using D'Agostino & Pearson test and an unpaired t-test (parametric) or Mann-Whitney test (non-parametric) were performed accordingly.

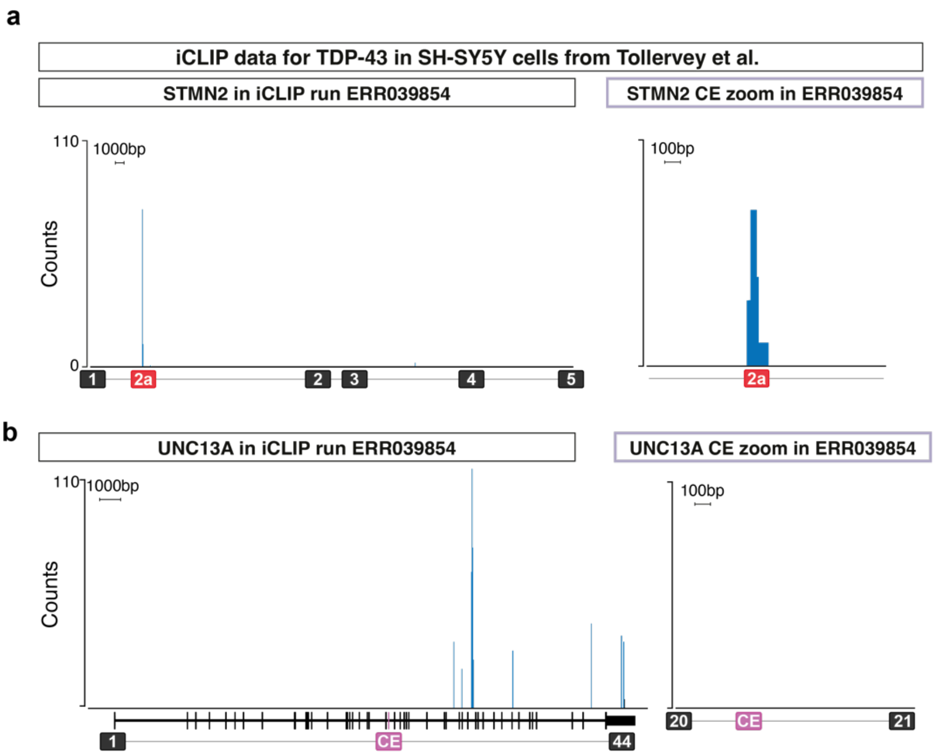

Supplementary Figure S4: TDP-43 binding to *STMN2* and *UNC13A* transcripts. (**a**-**b**) Gene browser track showing TDP-43 binding sites on *STMN2* (**a**) and *UNC13A* (**b**) pre-mRNAs determined by iCLIP for TDP-43 in human SH-SY5Y cells [9]. Results from the ERR039854 iCLIP run are represented showing both the full pre-mRNAs (left) and the region surrounding the *STMN2* and *UNC13A* cryptic exons (right).

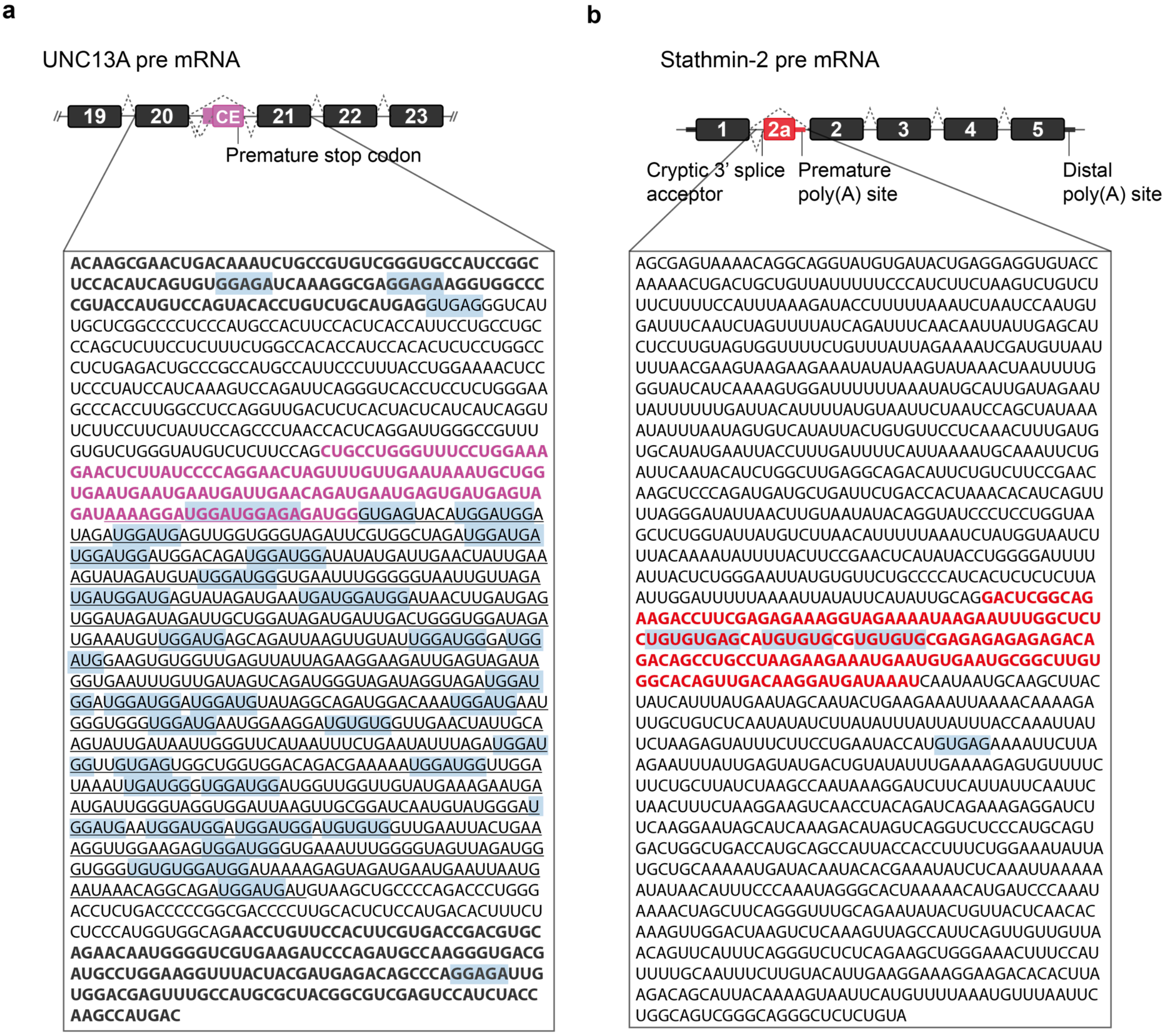

Supplementary Figure S5: TDP-43 binding motifs in *STMN2* and *UNC13A* transcripts. Sequence of *UNC13A* (**a**) and *STMN2* (**b**) pre-mRNAs, with the regions of TDP-43 binding identified by iCLIP [4, 9]. UG-rich motifs are highlighted in blue, and the sequence predicted to be bound by TDP-43 is underlined [4]. Cryptic exons are bolden in purple (*UNC13A*, **a**) and red (*STMN2*, **b**). Exons 20 and 21 of *UNC13A* transcript are bolded in gray (**a**).

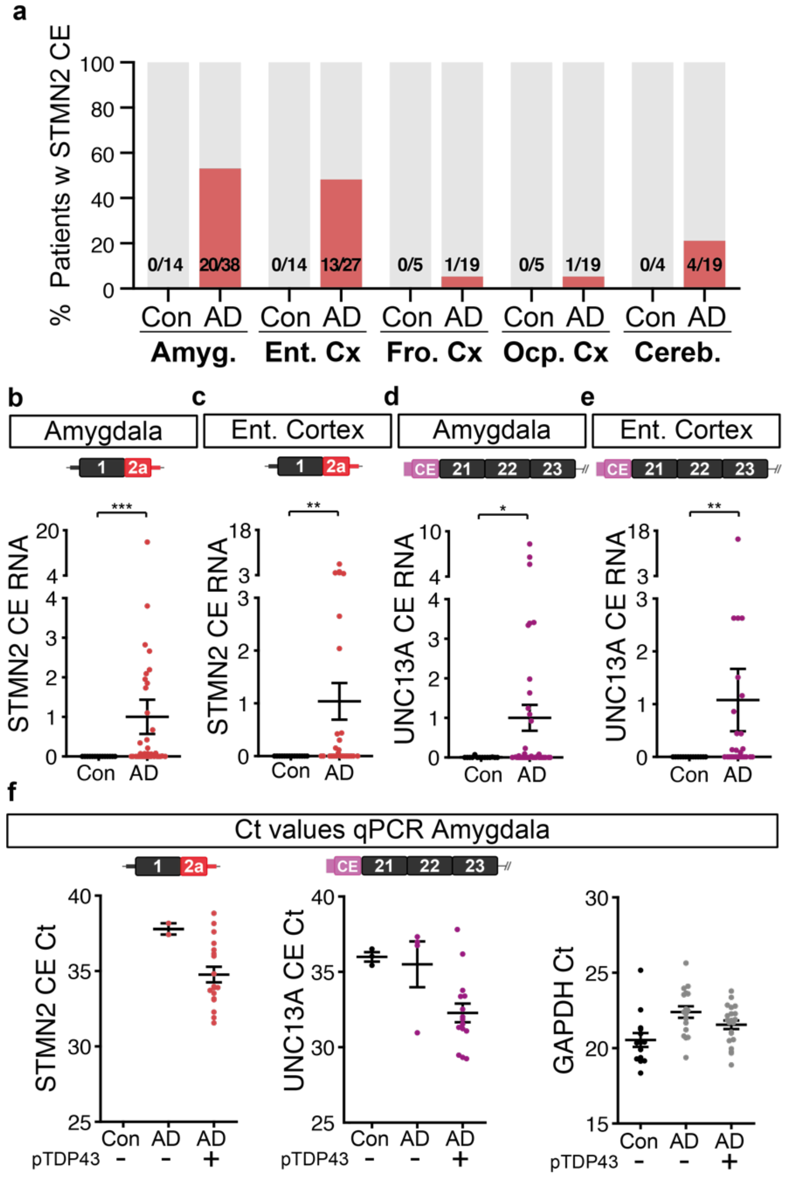

Supplementary Figure S6: Detection of *STMN2* and *UNC13A* cryptic exons in the amygdala and entorhinal cortex of post-mortem tissue from Alzheimer's disease patients. (**a**) Percentage of control or Alzheimer's disease patients in which *STMN2* cryptic exon was detected by qRT-PCR in different brain regions: amygdala (Amyg.), entorhinal cortex (Ent. Cx), frontal cortex (Fro. Cx), occipital cortex (Ocp. Cx) and cerebellum (Cereb.). Numbers of positive cases/total cases tested are indicated. Tissues were considered to have cryptic exons when an amplification curve was detected by qRT-PCR in all 3 triplicates. (**b-c**) Levels of truncated *STMN2* RNA (red) measured by qRT-PCR in post-mortem frozen amygdala (**b**) or enthorinal cortex (**c**) from control (Con) and patients with Alzheimer's disease (AD). (**d-e**) Levels of *UNC13A* cryptic exon (purple) measured by qRT-PCR in the amygdala (**d**) and entorhinal cortex (**e**). Data represent mean ± SEM, each dot represents results from individual patients. Normal distribution of data was tested using D'Agostino & Pearson test and an unpaired t-test (parametric) or Mann-Whitney test (non-parametric) were performed accordingly. (**f**) qRT-PCR cycle in which the levels of *STMN2* cryptic exon (red), *UNC13A* cryptic exon (purple) or *GAPDH* transcript (loading control, gray) reach the detection limit (Ct, cycle threshold), which inversely correlates with abundance of the target transcript in the amygdala of controls or Alzheimer's disease patients.

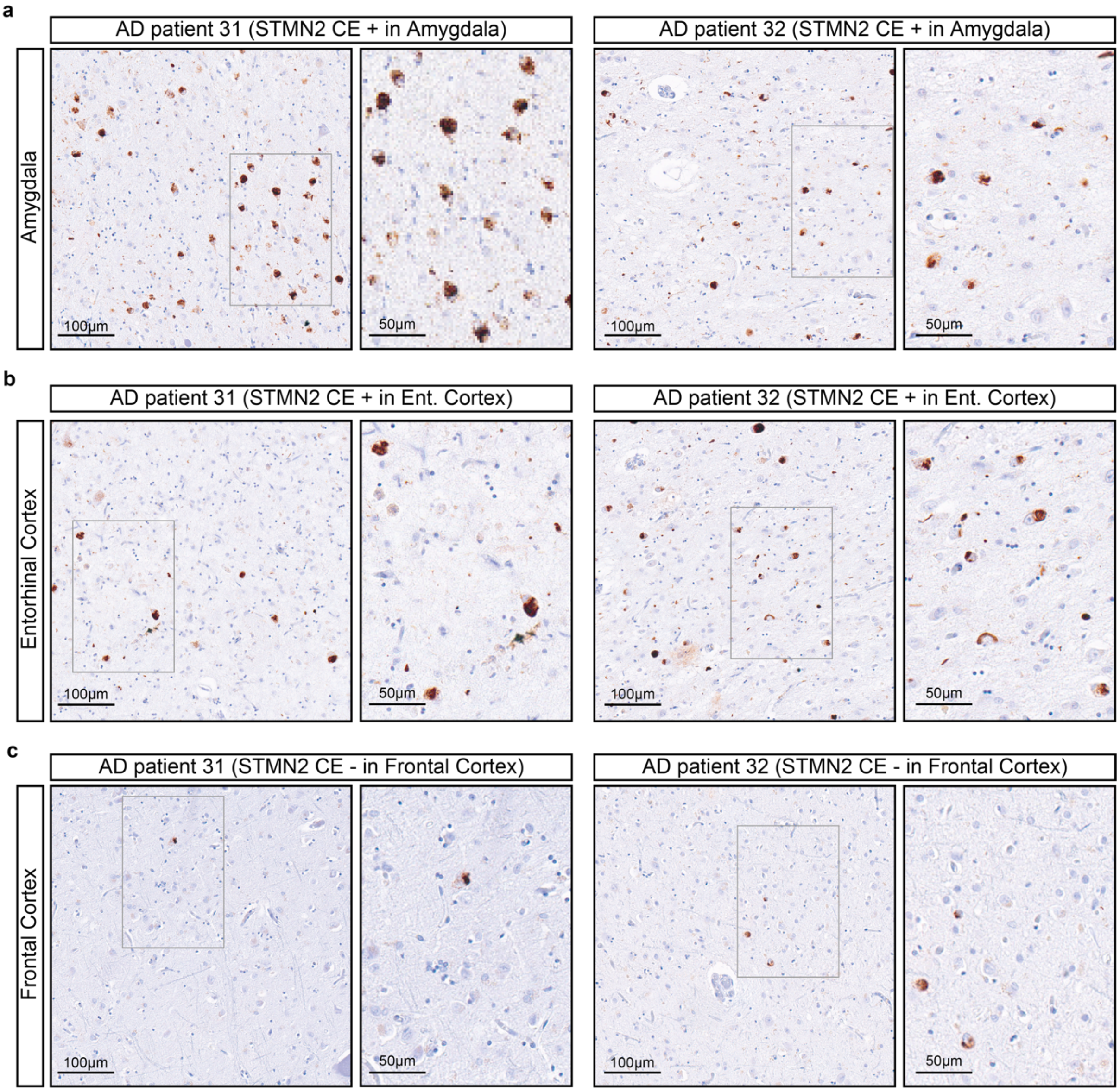

Supplementary Figure S7: TDP-43 pathology burden in different brain regions. Example micrographs of post-mortem brain tissue from Alzheimer's dementia patients 31 (left) and 32 (right) in the amygdala (**a**), entorhinal cortex (**b**) and frontal cortex (**c**), after immunohistochemical detection of phosphorylated TDP-43 (pTDP43). Scale bar is 100µm on the left images and 50µm on the insets.

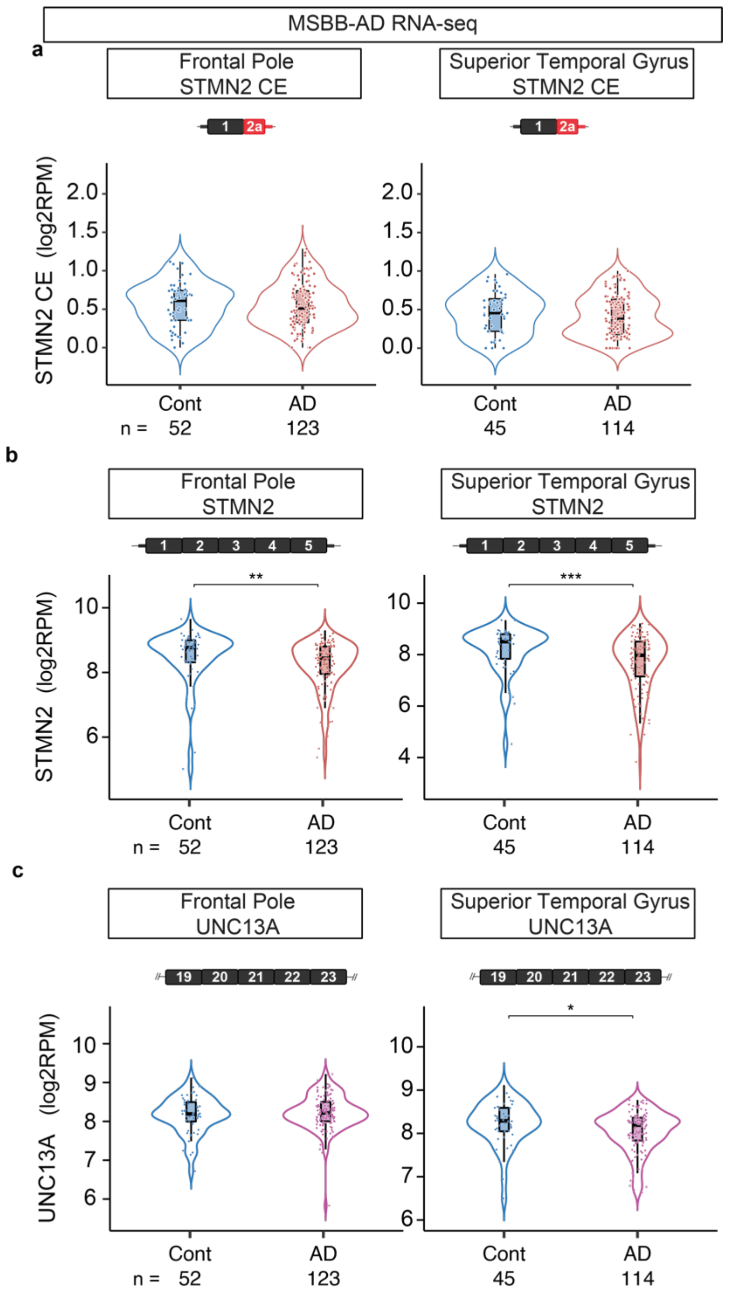

Supplementary Figure S8: RNA-seq analysis of *STMN2* and *UNC13A* mRNAs in frontal pole and superior temporal gyrus. (**a-b**) *STMN2* cryptic exon (**a**) and full-length (**b**), and *UNC13A* full-length (**c**) levels determined by RNA-seq analysis of the frontal pole and superior temporal gyrus from controls and Alzheimer's disease patients (data from Mount Sinai/JJ Peters VA Medical Center Brain Bank (MSBB-AD) [10]).

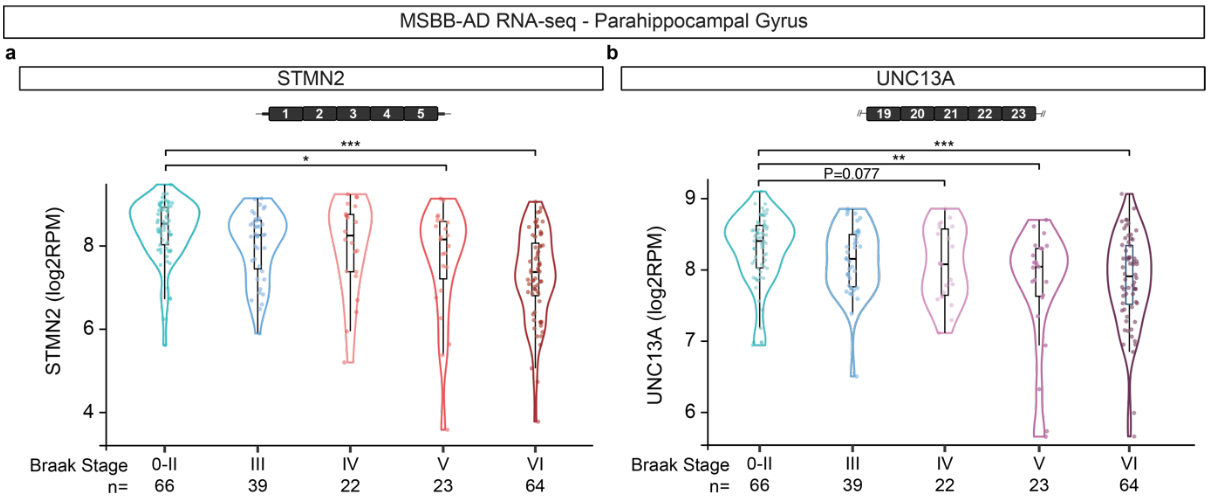

Supplementary Figure S9: RNA-seq analysis of *STMN2* and *UNC13A* mRNAs in parahippocampal gyrus with different Braak stages. (**a-b**) *STMN2* (**a**) and *UNC13A* (**b**) full-length RNA levels determined by RNA-seq analysis of parahippocampal gyrus from controls (Braak stages 0-II) and Alzheimer's disease patients classified according to their Braak stage (data from Mount Sinai/JJ Peters VA Medical Center Brain Bank (MSBB-AD) [10]).

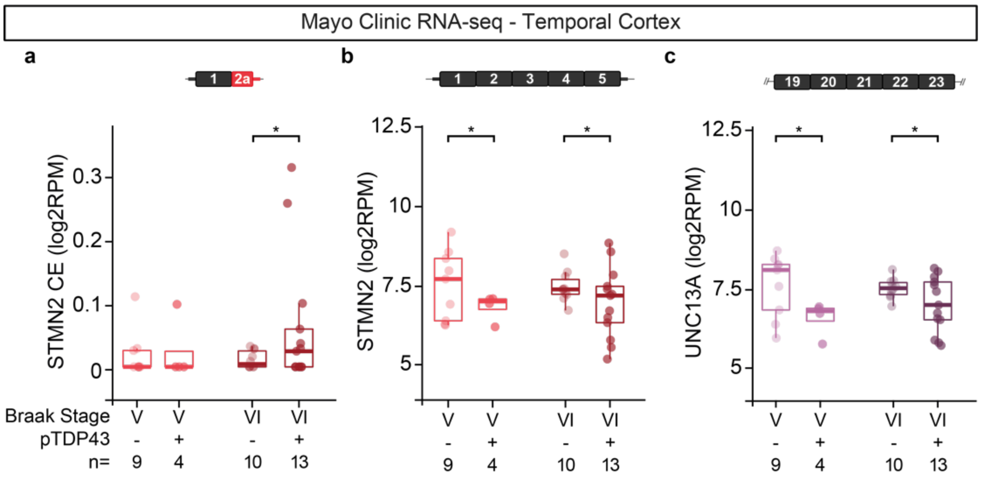

Supplementary Figure S10: RNA-seq analysis of *STMN2* and *UNC13A* mRNAs in Alzheimer's disease patients with Braak stages V and VI. (**a**-**c**) *STMN2* cryptic exon (**a**), and full-length *STMN2* (**b**) and *UNC13A* (**c**) transcript levels determined by RNA-seq analysis of temporal cortex from Alzheimer's disease patients with Braak stages V and VI. Brains with (+) and without (-) TDP-43 pathology were compared (data from Mayo Clinic [1, 2, 11]).

Supplementary Tables

Supplementary Table 1 – Probes and primers information.

Target	Application	Primer/Probe	Sequence (5'-3')	Ref.
Stathmin-2 Cryptic Exon	RT-PCR	Primer Fw	GCCTCTTGCTCTTTCTCTAGCACG	[8]
		Primer Rv	TTTCTCTCGAAGGTCTTCTG	[8]
Stathmin-2	qPCR	Primer Fw	AGCTGTCCATGCTGTCACTG	[3, 8]
		Primer Rv	GGTGGCTTCAAGATCAGCTC	[3, 8]
		Probe	ATTTGCTTCACTTCCATATCATCGTAAGTATAGATG	[3]
Stathmin-2 Cryptic Exon	qPCR	Primer Fw	CTTTCTCTAGCACGGTCCCAC	[3]
		Primer Rv	ATGCTCACACAGAGAGCCAAATTC	[3]
		Probe	CTCTCGAAGGTCTTCTGCCG	[3]
hnRNP L	qPCR	Primer Fw	GTGGAGATGGCTGATGGCTAC	
		Primer Rv	GCTCATCGCAGATCTCAAAGAAG	
UNC13A (Exons 19-20)	qPCR	Primer Fw	GGACGTGTGGTACAACCTGG	[7]
		Primer Rv	GTGTACTGGACATGGTACGGG	[7]
UNC13A (Exons 9-10)	qPCR	Primer Fw	CAGTGACTCCATGCACAGTTA	
		Primer Rv	TCAGCTGAGAGCTTCCCT	
UNC13A Cryptic Exon	qPCR & RT-PCR	Primer Fw	TGGATGGAGAGATGGAACCT	[7]
		Primer Rv	GGGCTGTCTCATCGTAGTAAAC	[7]
	qPCR	Probe	TTGGCATCTGGGATCTTCACGACC	
GAPDH	qPCR & RT-PCR	Primer Fw	GAAGGTGAAGGTCGGAGTC	[3, 8]
		Primer Rv	GAAGATGGTGATGGGATTTC	[3, 8]
	qPCR	Probe	CAAGCTTCCCGTTCTCAGCC	[3]

Supplementary Table 2 – Patient Information.

Subject	Condition	Age at death	Sex	Braak Stage
Cnt 1	Control	56	Female	0
Cnt 2	Control	76	Female	III
Cnt 3	Control	64	Female	N/A
Cnt 4	Control	85	Male	I
Cnt 5	Control	73	Male	0
Cnt 6	Control	68	Female	I
Cnt 7	Control	63	Male	0
Cnt 8	Control	>90	Female	II
Cnt 9	Control	77	Female	I
Cnt 10	Control	>90	Male	I
Cnt 11	Control	>90	Male	I
Cnt 12	Control	62	Male	0
Cnt 13	Control	78	Male	0
Cnt 14	Control	59	Male	0

Subject	Condition	Age at death	Sex	Braak Stage
Cnt 15	Control	>90	Male	I
AD 1	Alzheimer's disease	63	Male	VI
AD 2	Alzheimer's disease	61	Female	VI
AD 3	Alzheimer's disease	73	Female	VI
AD 4	Alzheimer's disease	58	Female	VI
AD 5	Alzheimer's disease	77	Female	VI
AD 6	Alzheimer's disease	77	Male	VI
AD 7	Alzheimer's disease	66	Female	VI
AD 8	Alzheimer's disease	>90	Male	VI
AD 9	Alzheimer's disease	78	Female	VI
AD 10	Alzheimer's disease	70	Male	VI
AD 11	Alzheimer's disease	66	Female	VI
AD 12	Alzheimer's disease	54	Female	VI
AD 13	Alzheimer's disease	70	Female	VI
AD 14	Alzheimer's disease	96	Female	V
AD 15	Alzheimer's disease	70	Male	VI
AD 16	Alzheimer's disease	73	Female	VI
AD 17	Alzheimer's disease	74	Male	V
AD 18	Alzheimer's disease	83	Female	VI
AD 19	Alzheimer's disease	80	Male	VI
AD 20	Alzheimer's disease	61	Female	VI
AD 21	Alzheimer's disease	73	Female	VI
AD 22	Alzheimer's disease	>90	Female	V
AD 23	Alzheimer's disease	80	Male	V
AD 24	Alzheimer's disease	82	Male	V
AD 25	Alzheimer's disease	74	Female	VI
AD 26	Alzheimer's disease	72	Female	VI
AD 27	Alzheimer's disease	97	Female	V
AD 28	Alzheimer's disease	74	Male	VI
AD 29	Alzheimer's disease	75	Male	VI
AD 30	Alzheimer's disease	86	Female	VI
AD 31	Alzheimer's disease	72	Female	VI
AD 32	Alzheimer's disease	87	Female	VI
AD 33	Alzheimer's disease	>90	Male	VI
AD 34	Alzheimer's disease	65	Male	VI
AD 35	Alzheimer's disease	76	Female	VI
AD 36	Alzheimer's disease	88	Female	VI
AD 37	Alzheimer's disease	>=90	Male	VI
AD 38	Alzheimer's disease	>=90	Male	V
AD 39	Alzheimer's disease	86	Male	VI

Supplementary Table 3 – Postmortem tissue analysis of TDP-43-associated pathological changes.

			Amygdala			Entorhinal Cortex		Frontal Cortex	Ocp. Cortex	Cereb.
**Subject**	**Condition**	**TDP43 stage**	**pTDP43**	**STMN2 CE**	**UNC13A CE**	**STMN2 CE**	**UNC13A CE**	**STMN2 CE**	**STMN2 CE**	**STMN2 CE**
Cnt 1	Control	0	No	0	0	0	0	NA	NA	NA
Cnt 2	Control	0	No	0	0	0	0	NA	NA	NA
Cnt 3	Control	0	No	0	0	0	0	NA	NA	NA
Cnt 4	Control	0	No	0	0	0	0	NA	NA	NA
Cnt 5	Control	0	No	0	0	0	0	NA	NA	NA
Cnt 6	Control	0	No	0	0	0	0	NA	NA	NA
Cnt 7	Control	0	No	0	0	0	0	NA	NA	NA
Cnt 8	Control	0	No	0	0	0	0	0	0	0
Cnt 9	Control	0	No	0	0	0	0	0	0	0
Cnt 10	Control	0	No	0	0	0	0	0	0	0
Cnt 11	Control	0	No	0	0	0	0	0	0	0
Cnt 12	Control	0	No	0	1	0	0	NA	NA	NA
Cnt 13	Control	0	No	0	1	0	0	NA	NA	NA
Cnt 14	Control	0	No	0	1	0	0	NA	NA	NA
Cnt 15	Control	0	No	NA	NA	NA	NA	0	0	NA
AD 1	Alzheimer	0	No	0	0	0	0	NA	NA	NA
AD 2	Alzheimer	0	No	0	0	0	0	NA	NA	NA
AD 3	Alzheimer	0	No	0	0	0	0	NA	NA	NA
AD 4	Alzheimer	0	No	0	0	0	0	0	0	0
AD 5	Alzheimer	0	No	0	0	0	0	0	0	0
AD 6	Alzheimer	0	No	0	0	0	0	0	0	0
AD 7	Alzheimer	0	No	0	0	0	0	0	0	0
AD 8	Alzheimer	0	No	0	0	0	0	0	0	0
AD 9	Alzheimer	0	No	0	0	NA	NA	NA	NA	NA
AD 10	Alzheimer	0	No	0	0	NA	NA	NA	NA	NA
AD 11	Alzheimer	0	No	0	0	NA	NA	NA	NA	NA
AD 12	Alzheimer	0	No	0	0	NA	NA	NA	NA	NA
AD 13	Alzheimer	0	No	0	0	NA	NA	NA	NA	NA
AD 14	Alzheimer	0	No	0	1	0	0	NA	NA	NA
AD 15	Alzheimer	0	No	0	1	NA	NA	NA	NA	NA
AD 16	Alzheimer	0	No	1	1	0	1	1	1	1
AD 17	Alzheimer	0	No	1	1	1	0	0	0	0
AD 18	Alzheimer	NA	Yes	0	0	0	0	NA	NA	NA
AD 19	Alzheimer	III	Yes	0	0	0	0	0	0	0
AD 20	Alzheimer	I	Yes	0	0	0	0	0	0	0
AD 21	Alzheimer	I	Yes	1	0	NA	NA	NA	NA	NA
AD 22	Alzheimer	II	Yes	1	0	1	1	0	0	0
AD 23	Alzheimer	III	Yes	1	0	1	1	0	0	0
AD 24	Alzheimer	NA	Yes	1	1	0	1	NA	NA	NA
AD 25	Alzheimer	NA	Yes	1	1	1	1	NA	NA	NA

			Amygdala			Entorhinal Cortex		Frontal Cortex	Ocp. Cortex	Cereb.
**Subject**	**Condition**	**TDP43 stage**	**pTDP43**	**STMN2 CE**	**UNC13A CE**	**STMN2 CE**	**UNC13A CE**	**STMN2 CE**	**STMN2 CE**	**STMN2 CE**
AD 26	Alzheimer	II	Yes	1	1	1	1	NA	NA	NA
AD 27	Alzheimer	NA	Yes	1	1	1	1	NA	NA	NA
AD 28	Alzheimer	II	Yes	1	1	1	0	0	0	0
AD 29	Alzheimer	II	Yes	1	1	1	1	0	0	1
AD 30	Alzheimer	II	Yes	1	1	1	1	0	0	1
AD 31	Alzheimer	III	Yes	1	1	1	1	0	0	0
AD 32	Alzheimer	III	Yes	1	1	1	1	0	0	0
AD 33	Alzheimer	II	Yes	1	1	1	1	0	0	0
AD 34	Alzheimer	II	Yes	1	1	1	1	0	0	0
AD 35	Alzheimer	NA	Yes	1	1	NA	NA	NA	NA	NA
AD 36	Alzheimer	I	Yes	1	1	NA	NA	NA	NA	NA
AD 37	Alzheimer	NA	Yes	1	1	NA	NA	NA	NA	NA
AD 38	Alzheimer	NA	Yes	1	1	NA	NA	NA	NA	NA
AD 39	Alzheimer	NA	Yes	NA	NA	NA	NA	0	0	0

References

1 Allen M, Carrasquillo MM, Funk C, Heavner BD, Zou F, Younkin CS, Burgess JD, Chai HS, Crook J, Eddy JA*, et al* (2016) Human whole genome genotype and transcriptome data for Alzheimer's and other neurodegenerative diseases. Sci Data 3: 160089 Doi 10.1038/sdata.2016.89

2 Allen M, Wang X, Burgess JD, Watzlawik J, Serie DJ, Younkin CS, Nguyen T, Malphrus KG, Lincoln S, Carrasquillo MM*, et al* (2018) Conserved brain myelination networks are altered in Alzheimer's and other neurodegenerative diseases. Alzheimers Dement 14: 352-366 Doi 10.1016/j.jalz.2017.09.012

3 Baughn MW, Melamed Z, Lopez-Erauskin J, Beccari MS, Ling K, Zuberi A, Presa M, Gonzalo-Gil E, Maimon R, Vazquez-Sanchez S*, et al* (2023) Mechanism of STMN2 cryptic splice-polyadenylation and its correction for TDP-43 proteinopathies. Science 379: 1140-1149 Doi 10.1126/science.abq5622

4 Brown AL, Wilkins OG, Keuss MJ, Hill SE, Zanovello M, Lee WC, Bampton A, Lee FCY, Masino L, Qi YA*, et al* (2022) TDP-43 loss and ALS-risk SNPs drive mis-splicing and depletion of UNC13A. Nature 603: 131-137 Doi 10.1038/s41586-022-04436-3

5 Krach F, Batra R, Wheeler EC, Vu AQ, Wang R, Hutt K, Rabin SJ, Baughn MW, Libby RT, Diaz-Garcia S*, et al* (2018) Transcriptome-pathology correlation identifies interplay between TDP-43 and the expression of its kinase CK1E in sporadic ALS. Acta Neuropathol 136: 405-423 Doi 10.1007/s00401-018-1870-7

6 Liu EY, Russ J, Cali CP, Phan JM, Amlie-Wolf A, Lee EB (2019) Loss of Nuclear TDP-43 Is Associated with Decondensation of LINE Retrotransposons. Cell Rep 27: 1409-1421 e1406 Doi 10.1016/j.celrep.2019.04.003

7 Ma XR, Prudencio M, Koike Y, Vatsavayai SC, Kim G, Harbinski F, Briner A, Rodriguez CM, Guo C, Akiyama T*, et al* (2022) TDP-43 represses cryptic exon inclusion in the FTD-ALS gene UNC13A. Nature 603: 124-130 Doi 10.1038/s41586-022-04424-7

8 Melamed Z, Lopez-Erauskin J, Baughn MW, Zhang O, Drenner K, Sun Y, Freyermuth F, McMahon MA, Beccari MS, Artates JW*, et al* (2019) Premature polyadenylation-mediated loss of stathmin-2 is a hallmark of TDP-43-dependent neurodegeneration. Nat Neurosci 22: 180-190 Doi 10.1038/s41593-018-0293-z

9 Tollervey JR, Curk T, Rogelj B, Briese M, Cereda M, Kayikci M, Konig J, Hortobagyi T, Nishimura AL, Zupunski V*, et al* (2011) Characterizing the RNA targets and position-dependent splicing regulation by TDP-43. Nat Neurosci 14: 452-458 Doi 10.1038/nn.2778

10 Wang M, Beckmann ND, Roussos P, Wang E, Zhou X, Wang Q, Ming C, Neff R, Ma W, Fullard JF*, et al* (2018) The Mount Sinai cohort of large-scale genomic, transcriptomic and proteomic data in Alzheimer's disease. Sci Data 5: 180185 Doi 10.1038/sdata.2018.185

11 Wang X, Allen M, Is O, Reddy JS, Tutor-New FQ, Castanedes Casey M, Carrasquillo MM, Oatman SR, Min Y, Asmann YW*, et al* (2022) Alzheimer's disease and progressive supranuclear palsy share similar transcriptomic changes in distinct brain regions. J Clin Invest 132: Doi 10.1172/JCI149904
